# Light-Induced Structural Evolutions in Electrostatic Nanoassemblies

**DOI:** 10.3390/polym18020190

**Published:** 2026-01-09

**Authors:** Mohit Agarwal, Ralf Schweins, Franziska Gröhn

**Affiliations:** 1 Department of Chemistry and Pharmacy, Interdisciplinary Center for Molecular Materials, Friedrich-Alexander Universität Erlangen-Nürnberg, Egerlandstr. 3, D-91058 Erlangen, Germany; mohit.agarwal@fau.de; 2 Institut Laue-Langevin, DS/LSS, 71 Avenue des Martyrs, F-38000 Grenoble, France; schweins@ill.eu

**Keywords:** electrostatic self-assembly, in situ SANS, kinetics, nanoparticle morphology evolution, spherical-to-ellipsoidal transition

## Abstract

Studying nanoscale self-assembly in real time using external stimuli unlocks new opportunities for dynamic and adaptive materials. While electrostatic self-assembly is well-established, real-time monitoring of its structural evolution under light irradiation remains largely unexploited. In this study, we employ light-responsive azobenzene dyes (Acid Yellow 38, AY38) and pH-sensitive polyamidoamine (PAMAM) dendrimers to investigate the kinetics of electrostatic self-assembly under UV irradiation. Using a custom in situ small-angle neutron scattering (SANS) setup, we track the real-time morphological transformations of self-assembled structures with sub-minute resolution. We introduce two distinct pathways: method A (pre-irradiated *cis*-AY38 for controlled, slow kinetics) and method B (direct UV-induced self-assembly, fast kinetics). The results reveal that *trans-cis* isomerization kinetics dictate the rate of self-assembly, influencing aggregate stability, *ζ*-potential evolution, and final morphology. Structural analysis using dynamic and static light scattering (DLS and SLS) and SANS elucidates a transition from spherical to ellipsoidal morphologies governed by electrostatic and dipole-dipole interactions. These findings establish photoisomerization-driven self-assembly as a robust mechanism for tunable nanoscale architectures, paving the way for adaptive photonic materials, targeted drug delivery, and reconfigurable nanostructures.

## 1. Introduction

Achieving precise control over nanoscale self-assembly is crucial for designing responsive materials with applications in drug delivery, nanophotonics, and soft robotics. Electrostatic self-assembly, which relies on non-covalent interactions between charged building blocks, offers a promising route to such materials [1,2,3,4,5,6]. Conventional methods, such as *pH* changes [7,8,9,10], ionic strength variations [11,12], or temperature shifts [13,14], enable control over self-assembly. However, these methods require chemical modifications, external additives, or often disadvantageous temperature increases, making them less practical for real-time, reversible applications [15,16,17,18]. Light-responsive self-assembly provides a non-invasive, reversible, and highly tunable alternative to traditional methods [18,19,20,21,22,23]. Among light-responsive molecules, azobenzene dyes are particularly promising because light switches their molecular geometry and polarity, enabling time-programmable self-assembly [24,25,26,27]. In 2010, we (Gröhn et al.) demonstrated for the first time a light-switchable particle size in nanoscale objects [4], in that case formed by electrostatic self-assembly.

Despite extensive studies on equilibrium structures on azobenzene-based assemblies, time-resolved investigations of light-induced self-assembly are extremely rare—and, to our knowledge, absent for electrostatically driven systems. Existing work primarily examines endpoint morphologies rather than real-time dynamics [18,28,29]. Understanding these temporal processes is essential for creating truly adaptive nanomaterials.

To address this gap, in this study we investigate the assembly of Acid Yellow 38 (AY38), a di-anionic sulfonate-functionalized azobenzene dye, and polyamidoamine (PAMAM) dendrimers, a class of pH-responsive cationic macromolecules, to elucidate the mechanism of light-controlled electrostatic self-assembly. AY38 azo dye offers a *cis*-enriched photostationary state upon irradiation and slow thermal back-isomerization, enabling time-resolved tracking of protocol-dependent restructuring [30,31].

Unlike previous investigations that demonstrated the size and morphology changes upon UV irradiation in azobenzene-based assemblies, with notable studies focusing on equilibrium states and endpoint measurements [6], we herein systematically track structural changes in real-time, providing quantitative insight into the role of charge ratio, dendrimer generation, and isomerization kinetics in controlling self-assembly dynamics. This study addresses this critical gap by developing a dedicated in situ small-angle neutron scattering (SANS) setup to capture the kinetics of electrostatic self-assembly under UV irradiation.

Recent studies have explored real-time in situ dynamic self-assembly using advanced spectroscopic and scattering techniques. Huang et al. used fluorescence spectrometry to monitor kinetically controlled self-assembly via optical transitions [32], while Jensen et al. utilized synchrotron SAXS to capture the entire micelle formation process [33,34,35]. Kelly et al. were among the first to integrate in situ UV-Vis and SANS to reveal reversible micellar transformations from wormlike micelles to fractal-like aggregates in response to photoisomerization [36]. Building on these insights into light-responsive micellar transformations, i.e., a light response based on the classical concept of micelle formation, our study expands this concept to electrostatic macroion-dye assemblies, elucidating how photoisomerization determines real-time morphological evolutions, captured here for the first time via in situ UV irradiation using SANS.

**Scheme 1:**
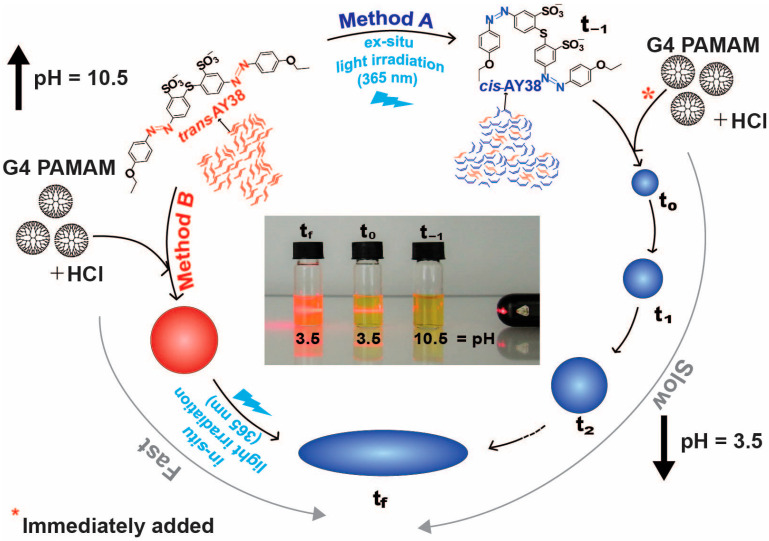
Representation of the two different pathways to prepare electrostatic assemblies of anionic azo dye molecules and cationic dendrimer macroions (Acid Yellow 38 and G4 PAMAM dendrimer). Method A (pre-irradiation) slows down the formation of assemblies by a factor of 10 compared to method B (post-irradiation). The light irradiation occurs at λ = 365 nm for 20 min in both methods. The picture in the middle represents the scattering of samples in method A at different times (t_−1_ = before the addition of acid, t_0_ = immediately after mixing the acid, t_f_ = after 3 h).

To investigate the mechanism of electrostatic self-assembly between a di-anionic AY38 and cationic PAMAM dendrimer macroions, this study leverages the azo dye’s photo switchability and the dendrimer’s *pH* responsiveness. Two distinct self-assembly pathways were designed to explore these dynamics, as shown in Scheme 1: method A (slow kinetics, ≈3 h) and method B (fast kinetics, <20 min). Method A slows restructuring to resolve intermediates, whereas method B captures fast endpoint evolution under in situ SANS measurements. The prolonged half-life of *cis*-AY38 (≈12 h) enables a detailed structural characterization using SANS, dynamic light scattering (DLS), static light scattering (SLS), and *ζ*-potential measurements. Due to its fast kinetics, an in situ approach was necessary to capture the transient structural states. This was achieved using a dedicated in situ setup integrated with the D11 instrument, as detailed in the experimental section.

To investigate the real-time morphological evolution of photoresponsive assemblies, we focused primarily on method A, which exploits the prolonged lifetime of the *cis* isomer to slow down assembly kinetics. This allows for a detailed characterization of intermediate structures and a clearer understanding of the underlying (re)structuring pathway. In contrast, method B assists in elucidating the relationship between photoisomerization rates and nanoscale organization. By addressing key gaps in the understanding of photoresponsive electrostatic self-assembly, we investigate how the isomeric states of the azobenzene dye influence the structure and dynamics of the resulting assemblies. Through this dual-method approach of method A (slow kinetics) and method B (fast kinetics), we establish a direct correlation between photoisomerization rates and nanoscale organization, offering a new paradigm for light-controlled nanostructure fabrication. These findings advance the understanding of photoisomerization-driven self-assembly and establish a framework for designing photoresponsive materials with applications in adaptive nanomaterials, optoelectronic devices, and targeted drug delivery systems.

## 2. Materials and Methods

### 2.1. Chemicals

Polyamidoamine dendrimers of generations 4 (10 wt. % in methanol) and 5 (5 wt. % in methanol) were purchased from Aldrich (Darmstadt, Germany). DLS confirmed the radius and size distribution given by the manufacturer, Dendritech (Midland, MI, USA). Acid Yellow 38 with >40% purity was purchased from Merck (Darmstadt, Germany) and purified to >98% by multiple recrystallizations using water and ethanol, as described previously [37,38]. D_2_O, NaOD, and DCl were purchased from Merck, Darmstadt, Germany, and NaOD and DCl were used to adjust the *pD* of the solution.

### 2.2. Sample Preparation

D_2_O was used as a solvent to prepare all samples, and all the reagents used throughout the study, except for the assembly components, were deuterated. The stock solutions of azo dye (1.4 *×* 10^−3^ mol L^−1^) and PAMAM dendrimers (4.0 *×* 10^−5^ mol L^−1^) were prepared with D_2_O solvent in basic medium (*pD* = 10.5). The samples were prepared based on their charge ratio, *l_c_*, which represents the ratio of anionic dye sulfonate groups to cationic amine groups. The standard solutions of NaOD and DCl were prepared at 1.0 *×* 10^−1^ mol L^−1^ concentration. The *pH* measurements were performed with a Mettler Toledo *pH* meter. The *pH* values were converted into *pD* values using the formula given [39,40,41,42]:

(1) pD=pH+0.41

In method A, the dye was pre-irradiated out of the neutron beam for 20 min at *λ* = 365 nm. An appropriate amount of dendrimer solution and 0.1 M DCl was mixed immediately to induce the assembly formation. The slow evolution of assemblies is monitored using various techniques, including dynamic and static light scattering, UV-Vis spectroscopy, *ζ*-potential, and small-angle neutron scattering.

In method B, an appropriate amount of azo dye stock solution was diluted with D_2_O, followed by mixing with the dendrimer solution under continuous stirring. Later, the appropriate amount of 0.1 M DCl is added to induce the assembly formation process. The sample was then irradiated using a novel, custom-built, state-of-the-art in situ setup built at the D11 beamline. The setup consists of a UV lamp emitting 365 nm light, which is focused on the sample cuvette using a convex lens. The UV lamp can be controlled via a long cable from outside the interlocked SANS sample area. In situ UV light illumination of the samples, while being exposed to the neutron beam, takes place for 20 min.

### 2.3. Light Scattering

Dynamic and static light scattering (DLS and SLS) measurements were carried out on an ALV CGS3 (ALV, Langen, Germany) instrument with an ALV 5004 correlator using a HeNe laser with a wavelength of *λ* = 632.8 nm and 23 mW output power. The DLS and SLS experiments were performed simultaneously within the angular range of 20° ≤ *θ* ≤ 150° with 5°-degree steps at room temperature (25 °C).

The Brownian motion of the assemblies in the solution was detected by the fluctuations in the scattered intensity at a given scattering angle as a function of time. The intensity-time autocorrelation function *g*_2_(*q*, *τ*) is converted into the electric field-time correlation function, *g*_1_(*q*, *τ*), using the Siegert relation [43]:

(2) $$\;\;g_{2}\left(q,\tau \right)=1+{\left[g_{1}\left(q,\tau \right)\right]}^{2}$$

where $\vec{q}$ is the scattering vector (momentum transfer), and *τ* is the shift in time. *g*_2_(*q*, *τ*) is:

(3) $$\;g_{2}\left(q,\tau \right)=\frac{\langle I\left(q,t\right)I\left(q,t+\tau \right)\rangle }{{\langle I\left(q,t\right)\rangle }^{2}}$$

which describes the correlation of the intensity at time *t =* 0 and *t* + *τ*, and *I*(*q*, *t*) is the scattered intensity at time *t*. The scattering vector $\vec{q}$, is related to the scattering angle *θ* as:

(4) $$q=\left|\vec{q}\right|=\frac{4\pi n_{D}}{\lambda }sin\left(\frac{\theta }{2}\right)$$

where *n_D_* is the refractive index of the solvent.

The electric field-time autocorrelation function, *g*_1_(*q*, *τ*), was further analyzed by a regularized Laplace transformation using the CONTIN algorithm provided by S. Provencher to obtain the distribution of relaxation times [44,45]. The apparent diffusion coefficient, *D_app_*, is calculated as given below:

(5) $$\;\;D_{app}=q^{-2}\tau^{-1}$$

Measurements covered a broad range of *q* by measuring a scattering angle of 20° ≤ *θ* ≤ 150° with 5-degree steps. In the case of an angular dependence, *D_app_* was extrapolated to *q* = 0 to yield *D*_0_. In the absence of an angular dependence, the average value of *D_app_* is taken as *D*_0_. From the latter, the hydrodynamic radius (*R_H_*) can be calculated using the Stokes-Einstein relationship:

(6) $$\;R_{H}=\frac{kT}{6\pi D_{0}}$$

where *k* is the Boltzmann constant, *T* is the absolute temperature in Kelvin, and *η* is the solvent viscosity.

Static light scattering was employed to determine the structural characteristics of the assemblies. By measuring the angular dependence of scattered light intensity, key parameters such as the radius of gyration, *R_G_* were extracted, providing insights into particle size, shape (using the ratio of *R_G_* and *R_H_*), and overall topology in solution. The Rayleigh ratio, Δ*R_θ_* is required to calculate the *R_G_* value. The Δ*R_θ_* is given using the formula:

(7) $${\Delta R}_{\theta }=KcM_{w}P\left(q\right)$$

where *c* is the mass concentration, *M_w_* is the weight-averaged molecular weight, *P*(*q*) is the form factor, and *K* is the optical constant. *K* is given by:

(8) $$K=\frac{4\pi^{2}}{\lambda_{0}^{4}N_{A}}{\left(n_{std}\frac{dn}{dc}\right)}^{2}$$

where *n_std_* is the refractive index of the standard (toluene) and *dn*/*dc* the refractive index increment. The form factor *P*(*q*) can be described by the Guinier approximation:

(9) $$P\left(q\right)\approx exp\left(\frac{-q^{2}R_{G}^{2}}{3}\right)$$

or the Zimm equation [46]:

(10) $$\frac{Kc}{∆R_{\theta }}=\frac{1}{M_{w}}\left(1+\frac{1}{3}q^{2}\langle R_{G}^{2}\rangle \right)+2A_{2}c$$

*A*_2_ is the second osmotic virial coefficient, which determines the solvent-solute interactions. In the case of ideal solutions, *A*_2_ is zero when polymer-polymer and polymer-solvent interactions are equal, and the polymer shows an ideal chain behavior. The Zimm and Guinier equations are only valid for *q* × *R_G_* ≤ 1.2 [47]. The ratio of *R_G_/R_H_* provides essential information on particle topology. While the hydrodynamic radius, *R_H_*, is the radius of a friction-equivalent sphere in a fluid, *R_G_* is the root-mean-square distance of a molecule’s mass points from its center of mass, which can be further interpreted using the value of the *R_G_*/*R_H_* ratio as the shape information of nanoparticles in the solution.

For further analysis, SLS and small-angle neutron scattering (SANS) data have been merged, providing an extended *q*-range for examining nanoparticles at a wide scale.

### 2.4. ζ-Potential Measurements

*ζ*-potential measurements were performed on a Zetasizer Nano ZS analyzer equipped with a 4mW HeNe laser, *λ* = 633 nm (Malvern Instruments Ltd., Malvern, UK). All the measurements were performed with 1 mL of sample volume in disposable folded capillary cells (DTS 1070) at room temperature with five replicated measurements.

This technique measures the electrophoretic mobility *μ_e_* of the dispersed particles, including a fluid layer in the surrounding medium. Using the Helmholtz-Smoluchowski equation, the value of *ζ*-potential is calculated as [48,49]:

(11) $$\zeta =\left(\mu_{e}\;*\;\eta \right)/\varepsilon_{rs}\varepsilon_{0}$$

with *μ_e_* the electrophoretic mobility of charged particles, *η* the viscosity of the solution, *ε_rs_* the dielectric constant of the electrolyte solution, and *ε*_0_ the electric permittivity of vacuum.

### 2.5. UV-Vis Spectroscopy

The absorbance spectra were recorded with a JASCO V-630 spectrometer (JASCO Corporation, Tokyo, Japan) using a 200 μL sample volume in 1 mm path length quartz cuvettes to quantify the amount of dye isomers and measure the dye concentration. Measurements were performed at room temperature (25 °C). The *cis*-to-*trans* isomerization rate constant of AY38 azo dye is determined using kinetic measurements with 5 min time slices over a total measurement time of 5 h. The rate constant is calculated assuming a first-order reaction as given:

(12) $$ln\left[A\right]=-Kt+{\ln\left[A\right]}_{0}$$

Here, *A*_0_ is the initial concentration of *cis*-moiety, *A* is the concentration of *cis* isomer at a given time t, and *K* is the rate constant of the reaction.

### 2.6. Small-Angle Neutron Scattering

SANS is a non-destructive technique that utilizes the unique properties of neutrons to analyze the size, shape, and spatial arrangements of nanoscale assemblies in solution formed, for example, by polyelectrolytes and organic counterions. SANS measurements were performed at the D11 beamline (doi:10.5291/ILL-DATA.9-11-2079, doi:10.5291/ILL-DATA.9-12-662) at the Institut Laue-Langevin, Grenoble, France.

The SANS technique is based on the determination of intensity *I* of the scattered neutrons as a function of the scattering angle (can also be converted into scattering vector *q* using Equation (4)). The scattered intensity *I*, is given as [50]:

(13) $$I\left(q\right)=\emptyset V_{NP}{\left({∆\rho }_{SLD}\right)}^{2}P\left(q\right)S\left(q\right)$$

where ∅ is the volume fraction, *V_NP_* is the particle volume, ${∆\rho }_{SLD}$ is the difference in scattering length densities between the solute and the solvent, and *P*(*q*), the particle form factor, corresponds to the particle shape. The structure factor *S*(*q*) represents the interparticle correlations, considered equal to 1 in our case, as the samples have been diluted such that no interparticle interactions are present. The form factor is the square of the normalized total scattering amplitude *F*(*q*).

For example, for a sphere with a radius *R*, the form factor amplitude *F*(*q*) is [47].

(14) $$F\left(q\right)=3\frac{sinqR-qRcosqR}{{\left(qR\right)}^{3}}$$

While for an ellipsoid, the amplitude *F*(*q*) is [51]:

(15) $$F\left(q\right)=∆\rho V\frac{3(\sin qr-qr\;cosqr)}{{\left(qr\right)}^{3}}$$

where

(16) $$r={\left[R_{e}^{2}{sin}^{2}\alpha +R_{p}^{2}cos\alpha \right]}^{\frac{1}{2}}$$

where *α* is the angle between the ellipsoidal axes and $V=(4/3)\pi R_{p}R_{e}^{2}$ is the volume of the ellipsoid, *R_p_* is the polar radius along the rotational axis, and *R_e_* is the equatorial radius perpendicular to the rotational axis of the ellipsoid.

**Scheme 2:**
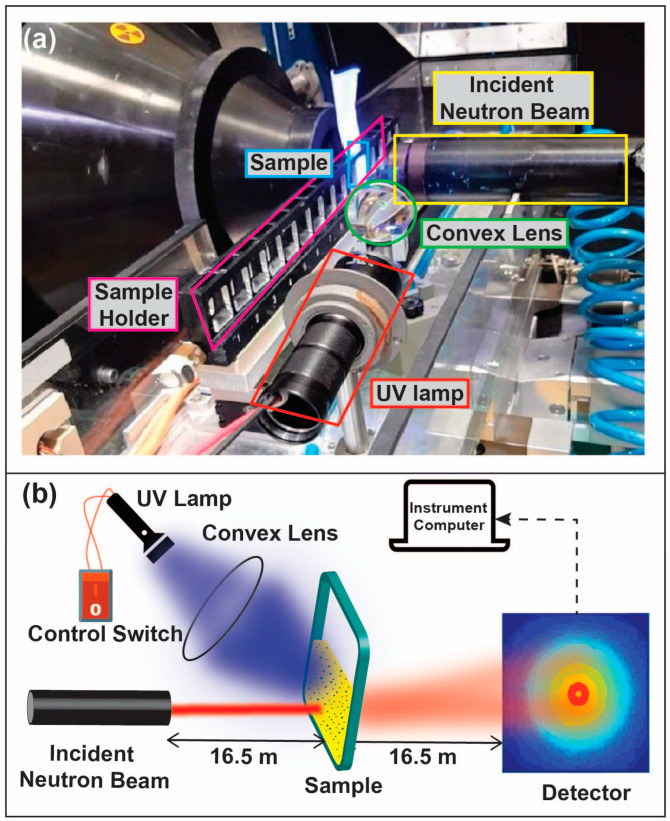
(**a**) Photograph and (**b**) schematic of the in situ UV irradiation setup used during SANS measurements. The UV lamp is positioned to ensure full illumination of the cuvette region intersected by the neutron beam (the irradiation geometry was kept fixed for all in situ experiments and thus does not substantially influence the outcome, as maximum irradiation is assured).

To investigate the effects of UV irradiation on the sample at a wavelength of λ = 365 nm, a custom in situ setup was designed, as illustrated in Scheme 2. The upper section of the figure shows a photo of the complete experimental setup. In contrast, the lower section provides a simplified schematic of the small-angle neutron scattering (SANS) apparatus under in situ UV irradiation. To replicate results obtained from static measurements, samples were exposed to UV light for a duration of 20 min, and SANS data were recorded simultaneously in the first 3 min with the scans of 10 s each and the next 17 min of 60-s time slices. Upon reaching the end of the irradiation, the scattering data did not vary much, and more than one short measurement could be added to make a single scattering curve. The sample environment was fully enclosed within a light-proof black cover during the SANS experiment to prevent unintended light exposure.

This in situ setup operates as an independent system controlled manually via a switch outside the sample chamber. A convex lens positioned between the UV lamp and the sample cuvette concentrates the UV light onto the sample, ensuring uniform illumination. During irradiation, the sample-to-detector and sample-to-collimation distances are fixed at 16.5 m to maintain consistent measurement conditions. This setup facilitates precise monitoring of structural changes induced by UV light in real time.

The UV lamp was positioned at an angle relative to the neutron beam to ensure that the sample volume probed by SANS is fully illuminated. (The angle was therefore chosen for geometrical/space constraints and was kept fixed for all in situ measurements towards the sample cuvette. The angle of these with the sample cuvette is approximately 30°. Under these conditions, the assembly behavior is governed by the UV irradiation and by maintaining consistent irradiation conditions across measurements.)

For experiments targeting slow kinetics, a sample-to-detector and collimation distance of 28 m was used in beamtime 9-12-662, while a configuration of 38 m with a collimation distance of 40.5 m was employed during beamtime 9-11-2079. These extended distances enabled the resolution of structural changes in assemblies over longer timescales (a total time of 3–4 h). In experiments with a 28 m sample-to-detector distance, 30 min time slices were taken, whereas in a 38 m distance, 1 min time slices were measured for the entire measurement duration. In contrast, a shorter sample-to-detector distance of 16.5 m with an equivalent collimation length was used for fast kinetic experiments, maximizing neutron flux to capture rapid structural transitions. The measured scattering vector ranges (*q*) measured were 0.018 nm^−1^ ≤ *q* ≤ 0.22 nm^−1^ for slow kinetics and 0.038 nm^−1^ ≤ *q* ≤ 0.58 nm^−1^ for fast kinetics. The scattering vector *q* is inversely related to the real-space length scale (*d*) by d=2π/q.

The 28 m configuration was selected for slow kinetics experiments to access lower q values (larger real-space length scales), which is essential to resolve the growth and restructuring of larger aggregates during the multi-hour evolution. Because the kinetics are slow, we could afford longer acquisition times (e.g., minute-to-30 min slicing depending on configuration), so the reduced flux associated with longer distances does not limit temporal resolution.

Samples for SANS were prepared in D_2_O to minimize incoherent background scattering from hydrogen. Measurements were conducted in Hellma 404-QX quartz cells (2 mm path length), with all samples containing AY38 dye at a concentration of c_AY38_ = 1 × 10^−4^ mol L^−1^. The experiments utilized a 15 mm diameter neutron beam with a wavelength λ = 6.0 Å (FWHM = 10%). A multi-tube ^3^He gas detector, consisting of a central panel with 192 horizontal tubes and two side panels with 32 vertical tubes each, provided a detection area of 256 × 256 pixels (tube length: 1 m with a diameter of 8 mm; pixel size: 4 × 8 mm^2^). Two-dimensional scattering data were corrected for empty cell scattering, electronic background, and detector non-uniformity using measurements of a 1 mm H_2_O standard. Transmission corrections were performed using Mantid 6.0 [52], and the data were normalized to absolute intensity using attenuated direct beam measurements specific to each instrument setup. Solvent and incoherent background scattering were subtracted from the azimuthally averaged scattering curves.

To extend the low *q* range for larger assemblies observed in slow kinetics, the SANS data were merged with static light scattering (SLS) data, yielding a combined *q* range of 0.0068 nm^−1^ ≤ *q* ≤ 0.22 nm^−1^. The scattering data were analyzed and modeled using the SasView 4.2.2 software, applying various form factors. The quality of the fit was evaluated using the reduced *χ*^2^ values (SasView 4.2.2: https://www.sasview.org/ (last accessed 16 September 2025)).

## 3. Results

To understand the dynamics of light-induced structural changes in self-assembly, we employed two distinct experimental pathways, each offering complementary insights into the role of azobenzene photoisomerization in driving nanoscale structural evolution. Method A utilizes pre-irradiated dye solutions to facilitate high-resolution, time-resolved structural characterization over extended timescales using SANS, dynamic light scattering (DLS), and *ζ*-potential measurements. In contrast, method B involves real-time UV irradiation of dye-dendrimer mixtures, enabling sub-minute resolution of the self-assembly process via an in situ UV-SANS setup.

### 3.1. Slow Kinetics (Method A)

#### 3.1.1. Light Scattering Measurements

To enable time-resolved structural analysis, we developed a modified sample preparation strategy referred to as method A that decelerates the assembly kinetics through pre-irradiation of the AY38 dye. UV irradiation converts ≈80% of the dye to its *cis* form, which, due to its sterically hindered geometry and lower dipole moment, binds less effectively to PAMAM dendrimers than the *trans* isomer [23]. As the dye gradually returns to the thermodynamically stable *trans* state (*cis* isomer half-life ≈ 12 h), the increasing population of *trans* isomers with higher dipole-dipole interaction strength and favorable binding geometry progressively drives aggregate formation [18,23]. This controlled, slow evolution enables detailed monitoring of assembly dynamics using SLS-SANS, DLS, and *ζ*-potential measurements.

These properties of AY38 are used in method A to slow down the growth of the aggregates. The dye was pre-irradiated, leading to a maximum *cis* dye amount, followed by the swift addition of dendrimer solution and acid. This leaves ≈ 20% of *trans*- and ≈ 80% of *cis* dye molecules to interact electrostatically with the dendrimer molecules and by dipole-dipole interactions with other dye molecules. Since *trans* isomers have a more suitable geometry and dipole moment, *trans*-molecules preferably bind with both dendrimers and other dye ions, while *cis* isomers primarily interact via electrostatic interactions with dendrimer molecules only [18,23]. Over time, the *cis* isomers gradually convert back to their *trans* state, thereby increasing the driving forces for assembly, such that the nanoassemblies grow.

**Figure 1:**
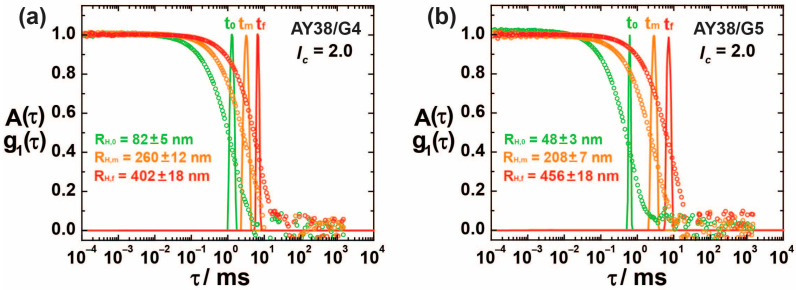
DLS data at a charge ratio (dye charges/dendrimer charges) of *l_c_* = 2.0 recorded at three key stages of the kinetics process: t_0_ ≈ 5 min (start, R_H,0_), t_m_ ≈ 60 min (midpoint, R_H,m_), and t_f_ ≈ 180 min (end R_H,f_). The plots display electric field autocorrelation *g*_1_(*τ*) (open circles) and the corresponding relaxation times distributions *A*(*τ*) (solid lines) at a scattering angle of *θ* = 90° for two dendrimer generations: (**a**) G4 and (**b**) G5. (The *R_H_* values are obtained by extrapolation to a 0° angle, and σ represents the particle size distribution at a 90° scattering angle, c_AY38_ = 1 × 10^−4^ mol L^−1^).

To gain deeper insight into the factors governing the self-assembly process, it is important to explore how the charge ratio of *l_c_* and dendrimer generation influences the kinetics and final morphology of the resulting aggregates. Figure 1 presents the results of angular-dependent dynamic light scattering in terms of the change in the *R_H_* values during the *cis*-to-*trans* (back) isomerization for assemblies formed with AY38 azo dye and two different generations of PAMAM dendrimers, generations 4 and 5 (G4 and G5), prepared using method A at a charge ratio of *l_c_* = 2.0. The experiment reveals three critical stages of the self-assembly process. Initially, at t_0_ ≈ 5 min, small particles with hydrodynamic radii R_H_ < 100 nm are observed. As the system evolves, a gradual increase in size is recorded, reaching R_H_ = 402 nm for AY38/G4 and R_H_ = 456 nm for AY38/G5 at t_f_ ≈ 180 min. This progression indicates a slow but continuous growth of the assemblies as *cis*-AY38 isomers convert to their more interacting *trans* state.

**Figure 2:**
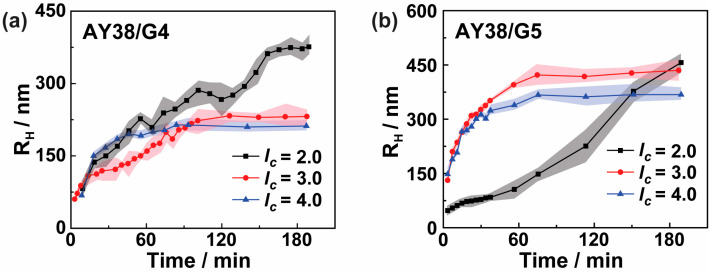
R_H_ obtained from multi-angle DLS on prolonged kinetics of multiple charge ratios (*l_c_* = 2.0, 3.0, and 4.0) using AY38 dye with (**a**) G4 and (**b**) G5 PAMAM dendrimers. The total duration for the kinetics was fixed at 3 h for all measurements. The different colored shaded areas represent the error bars obtained from several repeat measurements.

Figure 2 illustrates the kinetics profiles, where DLS measurements were recorded every 8–10 min. Higher charge ratios (*l_c_* ≥ 3.0) lead to smaller assemblies, while lower charge ratios (*l_c_* = 2.0) yield larger aggregates. This trend aligns with the prior observations in polyelectrolyte-dye systems, where the excess of dye charges enhances intermolecular attraction, leading to denser packing, even though those studies employed different preparation methods [4,18,23]. Similar charge-ratio-dependent behavior has been observed in cationic-anionic block copolymer assemblies, indicating that this principle may extend beyond azo-dye systems [9].

As Figure 2a displays, AY38/G4 assemblies at *l_c_* = 2.0 form aggregates with a stable size after 2.5 h. In contrast, at *l_c_* = 3.0, the aggregates stabilized in less than two hours, and at *l_c_* = 4.0, it took even less time (≈1.25 h) to form assemblies with a time-independent size. In contrast, with the G5 dendrimer, at *l_c_* = 2.0, aggregates with a stable size do not form within 3 h, but for *l_c_* = 3.0 and 4.0, stabilized aggregates build in 1–1.25 h. Thus, the G4 and G5 dendrimers exhibit a slow growth of the assemblies in the case of *l_c_* = 2.0, whereas the rate of aggregate size evolution increases with increasing *l_c_*. The assemblies stabilize quickly for higher charge ratios and do not aggregate further. Additionally, the assemblies grow the largest at *l_c_* = 2.0 as compared to the higher charge ratios where the stabilizing time was smaller.

#### 3.1.2. UV-Vis Spectroscopy

The different growth rates of assemblies at multiple charge ratios and with different PAMAM generations can be further understood in more detail by mapping the rate of back isomerization (i.e., rate constant, *k*/s^−1^) of the *cis* to *trans* state. The UV-Vis absorption was measured in 10 min intervals for a total duration of 10 h. The amounts of *cis* and *trans* isomers of the azo dye present in the sample solution were calculated [23]. The back isomerization process of AY38 azo dye is a first-order reaction. Using the amount of *cis* isomer and the first-order kinetics, the rate constant is calculated according to Equation (12).

**Figure 3:**
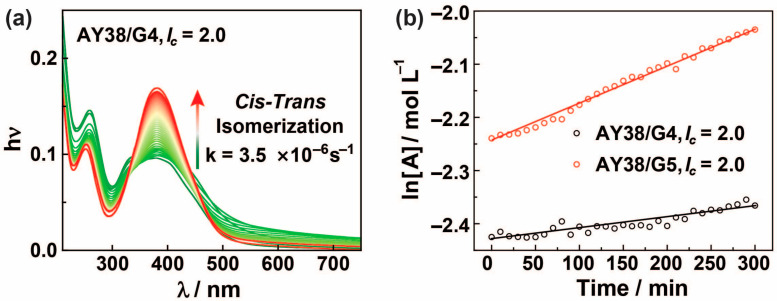
Slow kinetics of dye-dendrimer self-assembly characterized by UV-Vis absorption spectroscopy. (**a**) UV-Vis absorption spectra of AY38/G4 assemblies at *l_c_* = 2.0, recorded every 10 min over a 10 h period. The initial spectrum (dark green) corresponds to the pre-irradiated *cis*-AY38 dye. Upon mixing with dendrimer and acid, the dye gradually isomerizes back to the *trans* form. The isomerization rate constant (k) reflects the conversion kinetics. (**b**) First-order kinetic fits of *trans* isomer formation for AY38/G4 and AY38/G5 assemblies at *l_c_* = 2.0, based on Equation (12). On the y-axis, [A] represents the concentration of *trans* isomer in solution.

In Figure 3, the UV-Vis absorbance spectrum shows an immediate change upon mixing the pre-irradiated AY38 dye with the dendrimer and acid, indicating the beginning of *cis*-to-*trans* isomerization. Initially, approximately 80% of the dye is in the *cis* state, which gradually converts to the more thermodynamically stable *trans* isomer over several hours. To analyze the isomerization kinetics, absorbance measurements were recorded at regular 10 min intervals, and the decay of the *cis* isomer peak was tracked over time. The kinetic profile fits well to a first-order reaction model, which assumes that the rate of isomerization depends linearly on the concentration of the *cis* isomer. The rate constant *k* was determined to be 3.5 × 10^−6^ s^−1^ in the presence of dendrimer, significantly lower than the value observed for pure dye in solution (1.6 × 10^−5^ s^−1^). This reduced rate may arise from a photoprotective effect, wherein *cis*-AY38 bound to dendrimer molecules experiences restricted mobility and/or shielding, thereby slowing its relaxation to the *trans* state. Moreover, under excess dye conditions (*l_c_* ≥ 1.5), a surplus of unbound *cis* dye remains in solution, gradually isomerizing back to the *trans* state over time. The resulting increase in *trans* isomer concentration promotes secondary dipole–dipole interactions, which further drive the growth of electrostatic aggregates into larger nanostructures.

Regardless of the charge ratio and dendrimer generation, all systems in the present study exhibit approximately 80% of the dye in the *cis* state, which gradually undergoes back-isomerization to form *trans* dye molecules. This behavior contrasts with earlier studies, where variations in charge ratio and dendrimer generation significantly influenced dye-dendrimer interactions, which were discussed in the context of a different proportion of *cis* isomers in the solution [18,23,30]. The key difference lies in approach: in earlier studies dye-dendrimer aggregate formation occurred prior to irradiation under acidic conditions, and the change of the nano-object upon irradiation was observed. These preformed complexes limited photoisomerization efficiency due to constrained dye mobility. In contrast, the current approach maintains the dendrimers in an uncharged state, preventing aggregation while allowing unhindered isomerization dynamics of the dye upon irradiation. Only after irradiation is the acid added so that the pre-irradiated dye and the dendrimer form assemblies.

The results, summarized in Table 1, reveal a direct relationship between the rate constant of the back-isomerization and the growth rate of the assemblies. Stabilizing the assemblies correlates with the increasing formation of *trans* isomers in the solution. Due to a lower dipole-dipole moment, *cis* dye molecules exhibit minimal dye-dye interactions compared to their *trans* counterparts [23,31]. With their linear and extended structure, *trans* dye molecules offer a larger surface area for interaction with dendrimer molecules, maximizing electrostatic binding. This spatial configuration allows *trans* isomers to align more closely and orderly along the dendrimer surface, reducing steric hindrance and enabling dense packing. Furthermore, the higher dipole moment of *trans* dye molecules (27.96 Debye) compared to *cis* isomers (12.96 Debye) increases the likelihood of dye-dye interactions, enhancing their binding ability in electrostatic self-assemblies. The influence of dendrimer generation is also evident: assemblies formed with G4 dendrimers stabilize more slowly than those with G5, particularly at lower charge ratios. This difference likely stems from the increased number of terminal amine groups and higher surface charge density of G5 dendrimers, which provide more binding sites and stronger electrostatic attraction for the dye molecules. As a result, G5 dendrimers facilitate faster assembly formation compared to G4 dendrimers. This comparison highlights how dendrimer architecture and generation modulate the kinetics and final morphology of electrostatic self-assemblies.

#### 3.1.3. Small-Angle Neutron Scattering

**Figure 4:**
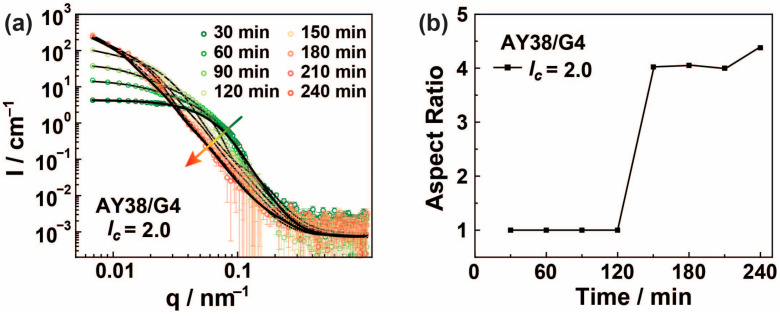
SANS on the long kinetics of the AY38/G4 system at *l_c_* = 2.0 (**a**) SLS-SANS data where both SLS and SANS measurements were performed separately on the same system with the same time intervals. The total duration of the experiment with both instruments was 4 h. In SANS, each measurement was performed at a 28 m sample-detector distance for 30 min. The color change from dark green to dark red represents the course of the kinetics. (**b**) The change in the aspect ratio (length/width) of the nano-objects resulting from structural model fitting as a function of time.

Small-angle neutron scattering (SANS) provides detailed structural insights into the slow kinetics of dye-dendrimer electrostatic assemblies. Figure 4a illustrates the evolution in the AY38/G4 system at *l_c_* = 2.0. The measurements were conducted over 4 h using the D11 instrument at a sample-detector distance of 28 m, with data collected in eight 30 min segments. The progression of the kinetics is represented by the color gradient from dark green to dark red. While the self-assembly process continues until stabilization, 30 min time slices were chosen due to the low sample count rate at the extended detector distance. After applying background corrections, the data were analyzed using various structural models (SasView 4.2.2 application), with all calculated parameters detailed in Table 2.

The scattering curves of the AY38/G4 system display distinct temporal evolution over 150 min, characterized by an increasing slope in the low q region, indicative of progressive particle growth. This observation aligns with the results from complementary light-scattering measurements, confirming a gradual increase in aggregate dimensions during the self-assembly process. The SANS data were best described by a spherical form factor during the first 120 min, corresponding to the initial growth phase where small, nearly isotropic particles (R ≈ 25 nm) gradually expanded to R ≈ 60 nm. Beyond this period, the scattering profile could no longer be satisfactorily fit with a spherical model, and an elongated ellipsoidal model provided a significantly improved fit (a comparison of spherical and ellipsoidal fits is shown with their residuals in Figure S1 and fitting details in Table S1 to confirm the fit quality of the measurement at 150 min). The transition occurred between 120 and 150 min, at which point the assemblies evolved into stable ellipsoidal structures with a semi-minor axis (R_ax_) of ≈ 35 nm and a semi-major axis (R_eq_) of ≈ 139 nm. The reduced *χ*^2^ values for all fits remained below 3.0, demonstrating excellent agreement between the experimental data and the applied models.

The corresponding aspect ratios (length/width) corresponding to the structural model fit results are illustrated in Figure 4b. A value of unity represents spherical geometry, whereas larger values indicate anisotropy. Remarkably, the aspect ratio increases sharply from ≈ 1 to ≈ 4 between 120 and 150 min, consistent with the morphological transformation from spherical to elongated ellipsoidal assemblies. This shape evolution reflects a reorganization process driven by the increasing population of *trans*-AY38 isomers over time. Possibly, as the more linear *trans* dye molecules gradually replace the bent *cis* isomers, the local packing within the dye-dendrimer complexes may become increasingly directional and could promote anisotropic association along one preferred axis, leading to elongation rather than isotropic growth. This anisotropic association favors elongation, while the electrostatic confinement by the dendrimer framework prevents unrestricted one-dimensional growth, yielding ellipsoids as the most energetically favorable intermediate morphology.

In previous studies employing method B, our group established the influence of charge ratio and dendrimer generations on the size and shape of electrostatic assemblies. [4,23] In the present work, we extend this investigation using method A to address the missing details in prior research, focusing on elucidating the evolution mechanism and the role of *cis-* and *trans* dye molecules in forming these assemblies. To explore the influence of charge ratio and dendrimer generations on the light response of electrostatic assembly using method A, the AY38/G5 system was used with *l_c_* = 2.0 and 3.0.

**Figure 5:**
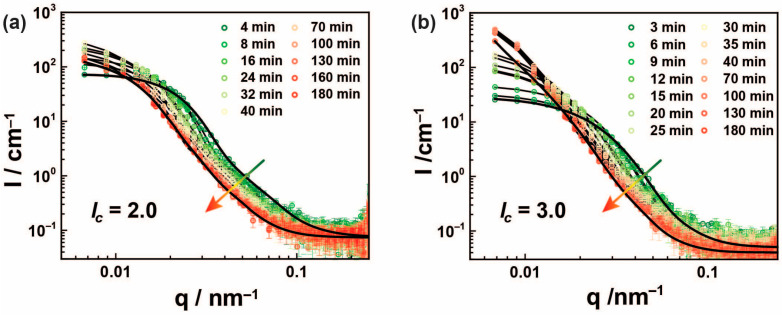
SLS-SANS on AY38/G5 long kinetics system at *l_c_*: (**a**) 2.0 and (**b**) 3.0. Both (SLS and SANS) measurements were performed separately on the same system at the same time intervals. The total duration of the experiment with both instruments was 3 h. SANS measurements were performed at a 38 m sample-detector distance. The color change from dark green to dark red represents the course of the kinetics.

Figure 5 displays the SANS measurements of AY38 dye with generation 5 PAMAM dendrimer under slow kinetics conditions. During the early stages of self-assembly, both charge ratios (*l_c_* = 2.0 and 3.0) exhibit a steady increase in the size of spherical structures (Table 3 and Table 4). For *l_c_* = 2.0, the radius of the particles reaches R = 87 nm within 4 min and increases to R = 105 nm over the following 4 min. The spherical morphology is maintained throughout the growth phase, with the radius gradually expanding to R = 176 nm by the 100 min mark, after which no further significant growth is observed. Although measurements continued for three hours, the assemblies remained stable in size beyond this point.

At *l_c_* = 3.0, smaller spherical particles were observed compared to those at *l_c_* = 2.0. This trend aligns with observations from post dye irradiation, where systems with higher charge ratios (*l_c_* > 1.0) form smaller assemblies than those with lower charge ratios [23]. The underlying mechanism in both the methods involves an excess of azo dye molecules, which contribute additional free sulfonate groups and thus increase the overall charge density of the system. Each *cis* dye molecule retains two negatively charged sulfonate groups, similar to its *trans* counterpart, but exhibits a lower dipole moment, which significantly reduces its propensity for dipole-dipole interactions. As a result, the *cis* isomers remain primarily engaged in electrostatic interactions with the positively charged PAMAM dendrimers, rather than aggregating among themselves. The presence of these surplus *cis* dye ions plays a crucial stabilizing role: they provide an abundance of accessible sulfonate charges, effectively saturating the available dendrimer binding sites and leading to the formation of compact, monodisperse assemblies.

**Figure 6:**
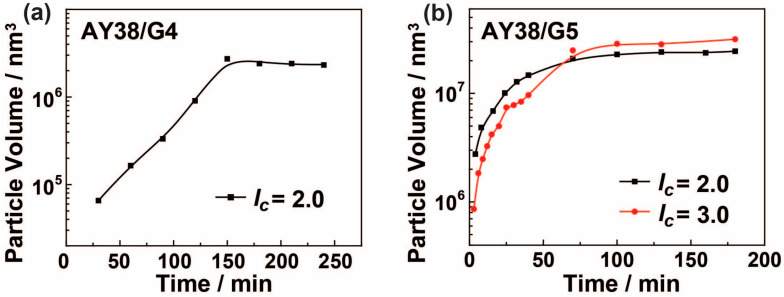
Time evolution of single-particle volume V(t) from SLS-SANS structural model fits for AY38/PAMAM assemblies (**a**) G4 at *l_c_* = 2.0 and (**b**) G5 at *l_c_* = 2.0 and 3.0. Volumes were calculated from the fitted dimensions using *V*_sphere_ = $\frac{4}{3}$*πR*^3^ and *V*_ellipsoid_ = $\frac{4}{3}$*π*abc (ellipsoid with rotational symmetry). G4 at *l_c_* = 2.0 shows a monotonic increase and plateau at ~150 min; G5 stabilizes earlier (≈100 min) at *l_c_* = 2.0 and 3.0, consistent with higher dendrimer valency and faster charge-compensation–driven growth. Lines are guides to the eye.

To compare growth across conditions when particle shapes evolve, we followed the single-particle geometric volume *V*(*t*) extracted from the SANS structural model fits (Figure 6). Volumes were computed using $V_{Sphere}=\frac{4}{3}\pi R^{3}$ during the spherical stage and $V_{Ellipsoid}=\frac{4}{3}\pi abc$ (axes a, b, and c from the ellipsoidal stage with rotational symmetry) after the sphere → ellipsoid transition. For G4 at *l_c_* = 2.0, *V*(*t*) increases monotonically and plateaus at around 150 min, coincident with the spherical → ellipsoidal morphology change and the aspect-ratio jump reported above in Figure 4b. In contrast, AY38/G5 reaches a volume plateau earlier, at *l_c_* = 2.0 and 3.0, with stabilization occurring at around 100 min, reflecting accelerated growth and earlier stabilization at higher generations. This generation-dependent kinetic acceleration is consistent with the higher valency of G5 (more charged amine groups) and more efficient charge compensation, which increases the capture rate of *trans*-AY38 and shortens the time required to reach electrostatic neutrality and structural stabilization. After the plateau has been reached, no further change in volume is observed within experimental uncertainty, indicating that an aggregation number increase via coalescence or continued unimer uptake may be negligible. Furthermore, from the SLS-SANS data, the forward-scattering intensity extrapolated to *q* → 0 (*I*_0_) can serve as an approximation for a relative aggregation number. It was determined and plotted versus time and versus SLS-SANS-derived particle volume in Figures S2 and S3. *I*_0_ versus time tracks the volume growth one-to-one and plateaus at the same time, confirming that the intensity-based aggregation metric is consistent with the particle volume (Figure S2). In contrast, however, towards the end of the kinetic *I*_0_ plateaus whereas volume keeps increasing as shown in Figure S3. As *I*_0_ approximately represents the aggregation number, it suggests that the particles do not grow in terms of aggregation number anymore but the particle volume increases; i.e., particles rearrange into a looser structure.

Although the SANS data were initially collected at 1 min intervals with 38 m sample-to-detector distance experiments, the smallest time segment presented in Figure 5 is 3 min. This adjustment was necessary due to two limiting factors: a low signal-to-noise (S/N) ratio and minimal discernible changes in scattering profiles at shorter intervals. To address these issues, data were consolidated into 3 min segments to improve S/N and ensure meaningful kinetic trends could be observed between consecutive measurements.

Another notable difference is the size distribution of assemblies. In previous studies, high charge ratio samples (*l_c_* ≥ 2.0) often exhibit bimodal distributions, possibly due to the predominance of *trans* dye molecules, which promote dipole-dipole interactions [23]. In contrast, the slow kinetics method starts with irradiated azo dye solutions dominated by *cis* isomers. The limited dye-dye interactions in this case result in the formation of only monomodal assemblies, eliminating any bimodal distribution characteristic.

#### 3.1.4. Charge Characteristics

**Figure 7:**
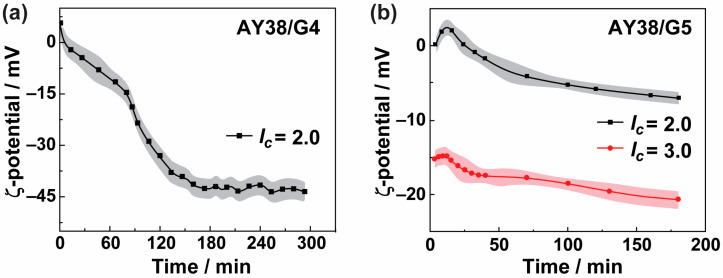
Time evolution of *ζ*-potential for AY38/PAMAM assemblies (**a**) G4 at *l_c_* = 2.0 and (**b**) G5 at *l_c_* = 2.0 and 3.0. The different colored shaded areas represent the error bars obtained from several repeated measurements.

Figure 7 presents the temporal evolution of the *ζ*-potential for assemblies formed via method A for AY38/G4 at *l_c_* = 2.0 and AY38/G5 at *l_c_* = 2.0 and *l_c_* = 3.0. Since the self-assembly is electrostatically driven, the *ζ*-potential provides a complementary readout of the assembly charge development helping to understand the particle size control. For AY38/G4 at *l_c_* = 2.0, *ζ* shifts from near-neutral values (≈0 mV) to strongly negative (≈−40 mV) over about 150 min, coinciding with the sphere → ellipsoid transition and the volume plateau (cf. Figure 6a). For AY38/G5 at *l_c_* = 2.0, *ζ* is also near neutral initially but reaches a stable negative value at around 100 min; at *l_c_* = 3.0, *ζ* is negative from the outset, reflecting excess anionic dye and stabilizes most rapidly. This behavior contrasts with post dye irradiation systems, where small, freshly formed aggregates exhibit strongly negative *ζ* and relax toward ≈ 0 mV as larger clusters form [4,18,23].

Under method A, pre-irradiation enriches *cis*-AY38, yielding weak initial binding; as *cis* → *trans* proceeds, the more linear, highly ordered *trans* isomers pack densely on the dendrimer, increasing the density of sulfonate groups on the dendrimer-dye assembly and driving *ζ* to more negative values. The earlier plateau for G5 and/or higher *l_c_* arises from higher dendrimer charges (more amine groups) and more efficient charge compensation, which accelerates dye uptake and halts further growth once electrostatic neutrality is approached.

The corresponding time of the *ζ* potential reaching a plateau as seen in Figure 7 with the volume plateau observed in Figure 6 demonstrates (which is clearly expressed for G4 but also indicated for G5) that charge accumulation and morphological changes occur and stop simultaneously. From this correlation, we infer that once electrostatic neutrality is approached, further aggregation ceases, defining the charge saturation threshold for structural stabilization. The inverse relationship between size (or particle volume/surface area) and *ζ*-potential underscores the interplay between size growth and charge stabilization (Figure S4). The steady decrease in *ζ*-potential likely reduces electrostatic repulsion between partially formed aggregates, enabling further association and enlargement. After approximately 3 h, both parameters, *ζ*-potential and particle volume, plateau, indicating that the system has reached an equilibrium state where isomerization-driven assembly is complete and no further structural reorganization occurs.

**Figure 8:**
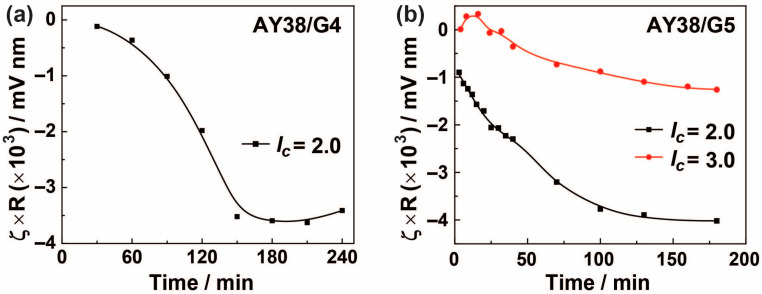
Time evolution of the effective (electrokinetic) particle charge *ζR* for AY38/PAMAM assemblies: (**a**) G4, *l_c_* = 2.0; (**b**) G5, *l_c_* = 2.0 and *l_c_* =3.0. The size (*R*) of the particles is taken from SANS fits: *R* = R_sphere_ for spherical particles and *R* = (abc)^1/3^ from the fitted ellipsoidal axes for anisotropic particles (shape equivalence by volume).

To understand the interplay of the particle charge characteristics with growth across changing morphologies, we track the effective (electrokinetic) charge *ζR* as a function of time as shown in Figure 8. Here, *R* is the particle size taken from the SANS fits, *R* = R_sphere_ during the spherical stage, and the equivalent-sphere radius R_eq_ = (abc)^1/3^ from the ellipsoidal volume thereafter to enable shape-independent comparison. For AY38/G4 at *l_c_* = 2.0, the charge increases from near zero to a stable negative plateau over about 150 min, coincident with the sphere → ellipsoid transition (Figure 4b) and the volume plateau (Figure 6a). AY38/G5 reaches a negative charge plateau earlier (≈120 min) at *l_c_* = 2.0 and earliest (≈100 min) at *l_c_* = 3.0, reflecting charge as a determining factor for assembly formation. The stronger (more negative) charge plateau at *l_c_* = 3.0, despite smaller final sizes, indicates denser anionic charge per particle, consistent with excess dye charges at higher charge ratios. Interpreted within standard electrokinetic models at fixed ionic strength, *ζ* reflects the electrostatic potential at the particle’s shear plane; multiplying by a characteristic length *R* provides a qualitative, effective charge indicator per particle. The temporal co-location of the charge plateau with the stabilization of volume and aspect ratio shows that morphological stabilization occurs once interfacial charge saturation is reached, even though *cis* → *trans* isomerization continues on longer timescales (cf. Figure 3).

**Figure 9:**
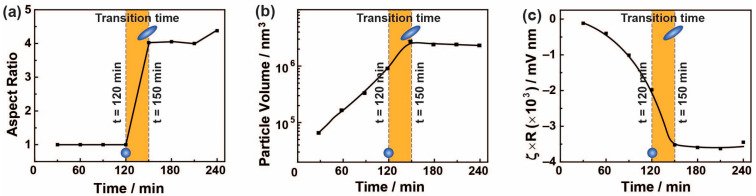
Coordinated transitions during the sphere → ellipsoid transformation for AY38/G4 at *l_c_* = 2.0. (**a**) Aspect ratio (length/width) from SANS fits (unity for spheres; > 1 for ellipsoids). (**b**) Single-particle volume, computed from best-fit SANS data. (**c**) Charge (*ζR*), tracking effective charge. The shaded band denotes the 120–150 min transition window.

Figure 9 shows the coordinated evolution of aspect ratio, single-particle volume, and effective charge (*ζR*) for AY38/G4 at *l_c_* = 2.0. All three quantities change synchronously between 120 and 150 min: the aspect ratio jumps from 1 to ≈ 4, the volume plateaus, and *ζR* reaches its inflection. This concurrence identifies the moment at which charge saturation halts further mass growth and initiates the sphere → ellipsoid transition. To visualize the coupling between morphology and interfacial charging, Figure 9 overlays three order parameters for AY38/G4 at *l_c_* = 2.0: the aspect ratio (length/width) from SANS fits, the single-particle geometric volume *V*(*t*), and the charge *ζR*. All traces change coordinately within the (120–150) min window: the aspect ratio jumps from 1 to ≥ 4, (*t*) approaches a plateau indicating that net mass uptake slows before shape fully arrests, and the charge reaches its plateau nearly concurrently. Together with Figure 6, Figure 7 and Figure 8, this establishes the kinetic sequence: (i) dye uptake and surface charge buildup suppress further growth (volume plateau), (ii) once a charge saturation threshold is crossed (*ζR* plateau), (iii) anisotropic packing of *trans*-AY38 drives the sphere → ellipsoid reorganization to completion, after which both aspect ratio and electrokinetic signal remain stable.

**Figure 10:**
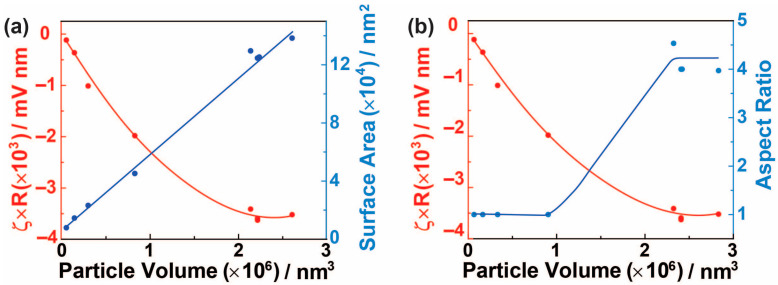
Coupling of particle volume V and particle surface area A with (**a**) particle charge *ζR* and (**b**) aspect ratio for AY38/G4 at *l_c_* = 2.0. The particle volume, surface area, and the *R* (in *ζ* × *R*) are taken from the SANS results.

Having identified the time and charge thresholds for the sphere → ellipsoid transformation, we further examine how particle volume (mass) and the particle surface co-evolve with particle charge and with aspect ratio as shown in Figure 10. The Figure 10a relates the single-particle volume V to both the surface area S and the charge (*ζR*) for AY38/G4 at *l_c_* = 2.0. Two regimes emerge. (i) Growth: volume and surface area increase together while charge becomes progressively more negative (stronger), reflecting dye uptake and interfacial charging. (ii) Reorganization: after the charge threshold is reached, the volume is essentially constant while the surface area continues to rise as assemblies convert from spheres to ellipsoids, consistent with the fact that spheres minimize surface area at a fixed volume. In Figure 10b, the co-location of the volume plateau with the *ζR* inflection and the rapid increase in aspect ratio indicates charge saturation-driven morphological arrest. Here, mass addition slows first, and then anisotropic packing of *trans*-AY38 increases interfacial area at near-constant volume until the ellipsoidal morphology stabilizes. Figure S5 shows the relation between charge and volume for AY38/G5 at *l_c_* = 2.0 and 3.0, where charge decreases similarity to the AY38/G4 system with an increase in the particle volume.

Hence, UV-Vis spectroscopy and *ζ*-potential measurements were used to monitor dye isomerization and the evolution of surface charge during assembly formation. Once ionic saturation is reached, excess dye molecules promote further aggregation through dipole–dipole interactions until the system reaches equilibrium. As demonstrated by light and neutron scattering data presented above, the assemblies typically cease growing in size and shape after 1 to 3 h, depending on the charge ratio and dendrimer generation. Notably, UV-Vis absorption measurements (Figure 3a) continued to show ongoing *cis*-to-*trans* isomerization over a 10 h period. This indicates that, despite continued isomerization, the assemblies reach a structurally stable equilibrium and no longer undergo further morphological changes. Importantly, the kinetics of assembly growth can be tuned by varying the charge ratio: lower charge ratios result in slower assembly growth, whereas higher charge ratios accelerate the process due to a greater availability of *trans* dye ions, which facilitates more efficient and stable binding with the dendrimer molecules.

### 3.2. Fast Kinetics (Method B)

While method A enables a detailed, time-resolved view of the restructuring process, real-world applications often demand faster responsiveness. To this end, we employed method B, a rapid in situ irradiation strategy (Scheme 2) that induces self-assembly within minutes. Although the fast kinetics make it challenging to resolve intermediate states, method B validates the endpoint structures and supports the mechanism inferred from method A. Comparing both approaches provides a holistic understanding of the system’s tunability, from slow, stepwise restructuring to rapid, responsive switching.

#### 3.2.1. Light Scattering

**Figure 11:**
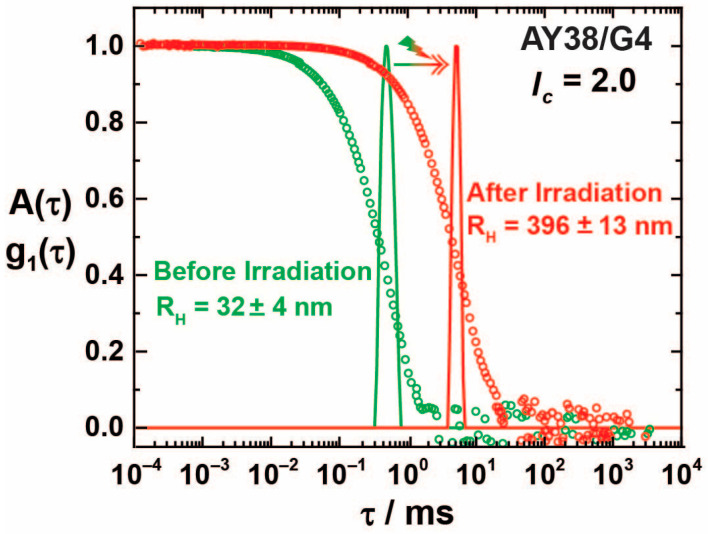
DLS of AY38/G4 system at *l_c_* = 2.0 before (green) and after irradiation (red): electric field autocorrelation *g*_1_(*τ*) (open circular symbols) and distribution of relaxation times *A*(*τ*) (lines) at a scattering angle of *θ* = 90°. (The *R_H_* values are obtained by extrapolation to a 0° angle, c_AY38_ = 1 × 10*^−^*^4^ mol L*^−^*^1^).

Figure 11 illustrates the change in size distribution upon irradiation in the AY38/G4 system at *l_c_* = 2.0 (preparation method B). The results are obtained using angular-dependent DLS, and the R_H_ values are calculated using Equation (6). Before irradiation, the nanoassemblies were *R_H_* = (32 ± 4) nm in size, increasing to more than 10-fold, R_H_ = (396 ± 13) nm upon irradiation. Willerich et al. observed a similar trend but a lower size evolution (≈5-fold) under slightly different conditions [4].

Using method B, the change caused by irradiation could be determined. However, the interest of the current study is to understand how the structures evolve and form while irradiating the sample and how the change in the degree of isomerization affects the structure. To the best of our knowledge, no prior work has systematically captured the formation dynamics of electrostatic assemblies under continuous UV irradiation. In situ monitoring using conventional techniques such as DLS, UV-Vis spectroscopy, or *ζ*-potential measurements poses significant technical challenges due to limitations in optical access and time resolution.

#### 3.2.2. Small-Angle Neutron Scattering

As discussed, SANS under in situ UV irradiation was developed at the D11 beamline (Scheme 2a). Additionally, before and after 20 min of irradiation (when the particles are in a stable state), an extended SANS and SLS scan (0.0046 nm^−1^ ≤ *q* ≤ 0.58 nm^−1^) has been performed to explore the whole structure of the nanoassemblies. The scan can take up to ≈ 1.5 to 2 h for the entire *q*-range. Whereas, to track the structural changes during irradiation, live SANS scans of 10 s each have been performed for the first 3 min and 60-s time slices for the next 17 min within a limited selected *q*-range (0.038 nm^−1^ ≤ *q* ≤ 0.58 nm^−1^) at a 16.5 m sample-detector distance.

**Figure 12:**
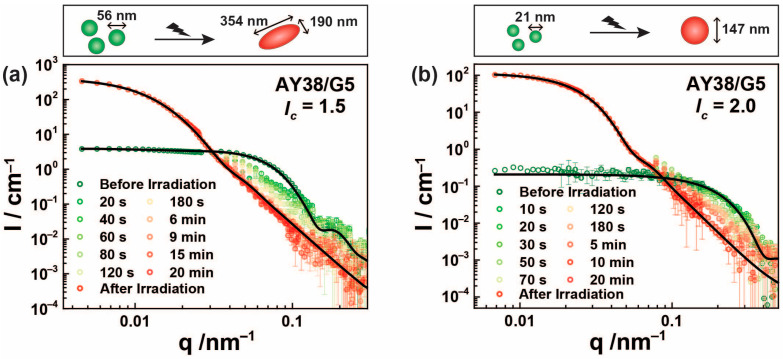
SANS of the AY38/G5 system at *l_c_* = (**a**) 1.5, and (**b**) 2.0 before, during, and after UV irradiation at λ = 365 nm using method B. Irradiation was applied for 20 min. The illustrations at the top of panels (**a**,**b**) represent the size and shape of the nanoassemblies before and after irradiation and correspond to the respective SANS data shown below. Each time-resolved SANS measurement was performed over 10-s intervals at a 16.5 m sample-to-detector distance and later summed when no further changes were observed across consecutive scans. To capture the complete structural profile, additional SANS and static light scattering (SLS) measurements were conducted separately on the same system before and after irradiation, once the assemblies had stabilized. These datasets were merged to extend the accessible *q*-range to 0.0046 nm^−1^ ≤ *q* ≤ 4.9 nm^−1^. SANS measurements were performed at sample-detector distances of 1.4 and 16.5 m.

Results for the AY38/G5 system at *l_c_* = 1.5 and 2.0 are shown in Figure 12. A quick evolution in the structure has been detected for up to 180 s, which then slows down later. This initial phase is likely driven by the fast *trans*-to-*cis* isomerization of AY38 under UV irradiation, which induces immediate changes in electrostatic interactions and triggers aggregation. As the irradiation continues, the system approaches a *cis*-enriched photostationary state, where the availability of *trans* dye molecules for further isomerization becomes limited, and the system begins to stabilize. Consequently, the rate of structural evolution slows down, transitioning into a more gradual reorganization process as the system asymptotically approaches a new equilibrium morphology. This biphasic behavior—initial rapid restructuring followed by a slower relaxation phase—is characteristic of many photoresponsive self-assembly systems and reflects the kinetic limitations imposed by the isomerization dynamics [35,36]. A transition from small aggregates to larger assemblies is indicated by the quickly declining plateau at the mid-*q* slope (0.038 nm^−1^ ≤ *q* ≤ 0.060 nm^−1^), which increases from *q*^0^ to *q*^4^ over time. Although the full structure cannot be predicted with this limited *q*-range (0.038 nm^−1^ ≤ *q* ≤ 0.58 nm^−1^), the particles certainly grow over time, as also confirmed by the end product in DLS studies.

SANS helps in understanding the growth mechanism of the electrostatic assemblies. In Figure 12a,b, a substantial difference in size and shape can be observed, caused by irradiation. At *l_c_* = 1.5, small spherically shaped nanoparticles of diameter 56 nm convert to ellipsoidal assemblies with an axial length of 190 nm and an equatorial length of 395 nm with a low polydispersity (σ ≤ 0.10) due to irradiation. At *l_c_* = 2.0, nanoparticle spheres of 21 nm convert into more than seven times larger spherical particles of 147 nm size. However, prior to irradiation, a few data points (obtained from SLS measurements) at low *q* do not fall within the range of a quality fit, which indicates traces of large aggregates coexisting with the small spherical particles.

**Figure 13:**
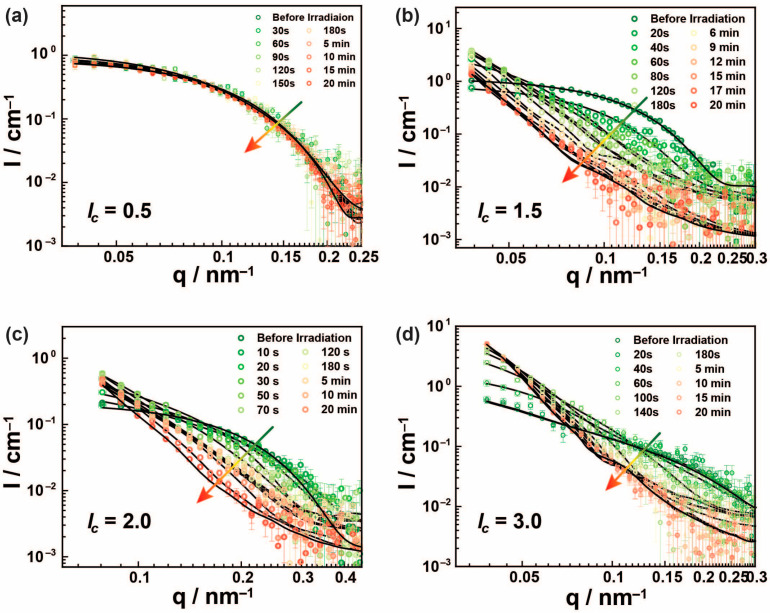
Short kinetics SANS results with their fitted data of the AY38/G5 system at *l_c_* = (**a**) 0.5, (**b**) 1.5, (**c**) 2.0, and (**d**) 3.0. The colored arrows in the SANS plots represent the course of the dynamics.

Figure 13 presents the short-timescale SANS data for the AY38/G5 system at *l_c_* = 0.5, 1.5, 2.0, and 3.0, along with their corresponding fits (particle volume is plotted as a function of time for the AY38/G5 system at *l_c_* = 0.5, 1.5, 2.0, and 3.0 in Figure S6, and the detailed parameter information is provided in Tables S2–S5). As noted in our previous studies, an excess of positive charges from the dendrimers stabilizes the dye-dendrimer assemblies at *l_c_* = 0.5. Consequently, no structural changes are observed upon irradiation in Figure 13a [4,23]. In contrast, for *l_c_* ≥ 1.5, significant changes in the scattering profiles are evident. These changes result from the excess dye ions that engage in dipole–dipole interactions. Upon UV irradiation, loosely bound dye ions partially dissociate from the assemblies. This weakens the electrostatic stabilization and leads to reduced particle stability. As a result, larger aggregates begin to form progressively over the course of the kinetics experiment.

The in situ SANS measurements under UV irradiation provide valuable insights into particle growth by self-assembly. The initial rapid increase in scattering intensity and the plateau behavior suggest a rapid assembly growth. The minimal changes in the scattering data observed beyond 180 s indicate a subsequent slower growth stage, revealing a two-step assembly process characterized by rapid initial aggregation followed by gradual structural evolution. However, the limited neutron flux on SANS instruments and large particle size (≈300–400 nm) do not allow for measuring the extended low *q*-range in a time-resolved manner as would be needed to identify the full assembly structures. Consequently, a full structural characterization using SANS is limited to the beginning and end of the irradiation period, where the sample remains stable and the full *q*-range can be effectively measured. In principle, this limitation could be mitigated by either increasing the concentration of the building blocks, thereby enhancing the scattering signal, or by slowing down the assembly process to allow adequate data collection within the accessible *q*-range. However, raising the concentration is not a viable option here, as it would introduce unwanted concentration-dependent effects on the self-assembly pathway [23].

## 4. Discussion

By integrating in situ SANS, multi-angle DLS and SLS, UV-Vis spectroscopy, and *ζ*-potential measurements, this study provides a mechanistic understanding of the dynamic (re)structuring process in photoresponsive dye-dendrimer assemblies in real time. The results demonstrate that photoisomerization kinetics directly dictate self-assembly dynamics. Across conditions, the kinetics follow a two-regime sequence: (i) a growth regime, in which particle volume *V*(*t*) increases monotonically with nearly monodisperse size distributions (DLS σ ≤ 10%) and *ζ* becomes progressively more negative, and (ii) a reorganization regime, in which *V*(*t*) has essentially plateaued while shape evolves from spheres to ellipsoids and charge *ζR* reaches an inflection/plateau (Figure 4, Figure 5, Figure 6, Figure 7 and Figure 8).

The juxtaposition in Figure 9 shows a threshold behavior: the sphere → ellipsoid transition initiates once the charge trace crosses its inflection point, after which the aspect ratio rises sharply while the volume remains nearly constant. At the molecular level, this sequence reflects the increasing *cis* → *trans* isomerization (or slow thermal back-isomerization in method A). *Cis*-rich states exhibit weaker, less ordered binding and low initial surface coverage. As *trans*-AY38 accumulates, the more linear, highly ordered isomers promote denser, directional packing on the dendrimer, increasing the surface density of sulfonate groups and driving *ζ* to more negative values. Once a charge saturation threshold is reached (Figure 9), further mass uptake is suppressed (volume plateau), and anisotropic packing of *trans*-AY38 completes the sphere → ellipsoid reorganization.

The dendrimer generation and charge ratio *l_c_* tune both the timescale and the endpoint of this pathway. G5 (higher valency) reaches electrokinetic plateaus and structural arrest earlier than G4, and higher *l_c_* accelerates stabilization further (Figure 6, Figure 7 and Figure 8). Mechanistically, a larger number of amine groups and/or greater dye excess enhance charge compensation and capture rates, allowing the system to attain the charge threshold sooner and to stabilize at smaller final sizes. Conversely, lower *l_c_* delays charge saturation and favors larger, more extended assemblies. Importantly, the kinetic protocol matters: under slow kinetics (method A) conditions, *ζ* evolves from near-neutral to strongly negative as *trans* content builds; in contrast, fast kinetics (method B) yields the opposite *ζ* trend at early times, underscoring that pathway dependence arises from how isomerization, adsorption, and growth are temporally coupled.

Together, these results move beyond endpoint characterization to deliver a predictive, process-aware framework: by jointly monitoring *V*(*t*), aspect ratio, and charge *ζR*, one can identify (i) a diffusion-controlled uptake regime, (ii) a charge saturation threshold that precipitates morphological change, and (iii) a shape-locking regime governed by a balance between electrostatic repulsion and dipole–dipole attraction. Table 5 and Table 6 summarize method sensitivities and the morphology map versus generation and *l_c_*. This framework enables temporal programming of structure tuning, not only of the final morphology but also of when and how it is reached via light-controlled modulation of isomer populations and interfacial charge. While full reversibility and deliberate capture of intermediate states remain challenges, the demonstrated control over the restructuring pathway itself opens avenues for time-encoded delivery platforms, reconfigurable soft materials, and other stimuli-responsive nanotechnologies.

## 5. Conclusions

This work establishes a direct, time-resolved link between photoisomerization kinetics and self-assembly dynamics in AY38/PAMAM systems. By combining in situ SANS with multi-angle DLS and SLS, UV-Vis, and *ζ*-potential measurements, it shows that light-controlled *cis* → *trans* conversion governs both when and how structure forms: particle volume grows first, an electrokinetic charge threshold is reached, and only then does a sphere → ellipsoid reorganization complete. The result is a mechanistic picture in which the interplay of photoisomerization and electrostatic interactions determines the resulting morphology.

Two kinetic pathways underscore the role of the protocol employed. Method A (slow kinetics; pre-irradiated *cis*, then assembly) spreads growth and restructuring over hours, enabling detailed mapping of thresholds and transitions, while Method B (fast kinetics; in situ irradiation) yields rapid aggregation and switching (≈20 min). System parameters tune both rate and endpoint: higher charge ratio (*l_c_* ≥ 3.0) and higher dendrimer generation accelerate charge compensation, advance the onset of shape change, and stabilize smaller aggregates; a lower *l_c_* prolongs growth and favors larger, more extended assemblies. Across conditions, the observed sphere → ellipsoid transition confirms that electrostatics coupled with dipole–dipole interactions, regulates morphology.

Together, these findings move beyond endpoint characterization to provide a process-aware design framework for adaptive nanomaterials: morphology and timescale of response can be programmed by modulating isomer populations and interfacial charge in real time. The approach is broadly transferable, and future work can extend it to alternative photochromes, ionic strengths, and solvent environments to refine control over light-mediated self-assembly and to realize temporally encoded functions in delivery, reconfigurable soft matter, and other stimuli-responsive technologies.

## Figures and Tables

**Table 1:** Rate constant, *k* (s^−1^), as calculated from UV-Vis spectroscopy for prolonged kinetics at different charge ratios (*l_c_* = 2.0, 3.0, and 4.0) for G4 and G5 PAMAM dendrimer—AY38 dye assemblies. The back isomerization process (*cis* to *trans* state) is considered a first-order reaction to calculate *k*. The overall uncertainty in calculating *k* is within 10%.

*l_c_*	Rate Constant, *k*/s^−1^	
	AY38/G4	AY38/G5
2.0	3.5 × 10^−6^	2.0 × 10^−6^
3.0	9.7 × 10^−6^	1.2 × 10^−5^
4.0	1.0 × 10^−5^	1.3 × 10^−5^

**Table 2:** SANS results: structural model fit parameters of AY38/G4 prolonged kinetics at *l_c_* = 2.0 (as shown in Figure 4a), obtained in SasView 4.2.2 using a polydisperse sphere model (30–120 min) and a polydisperse prolate ellipsoid model (150–240 min). The radius of the spherical particles is given as R_min_; in the case of ellipsoidal particles, R_min_ shows two axial radii_,_ and R_maj_ shows the equatorial radius. Polydispersity in size is given as PDI. The reduced *χ*^2^ shows the quality of the fit.

Time/min	Shape	R_min_/nm	PDI (R_min_)	R_maj_/nm	PDI (R_maj_)	*χ* ^2^
30	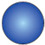	25	0.25			2.9
60	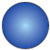	34	0.20			2.4
90	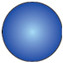	43	0.23			2.6
120	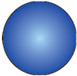	60	0.29			2.8
150	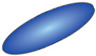	35	0.30	139	0.02	2.0
180	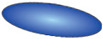	33	0.40	132	0.07	1.7
210	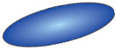	33	0.40	132	0.10	2.2
240	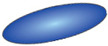	30	0.40	136	0.03	1.9

**Table 3:** SANS results: structural model fit parameters of AY38/G5 prolonged kinetics at *l_c_* = 2.0 (as shown in Figure 5a). The polydispersity of the spherical particles in size is given as PDI. The reduced *χ*^2^ shows the quality of the fit.

Time/min	Shape	Radius/nm	PDI	*χ* ^2^
4	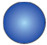	87	0.24	1.5
8	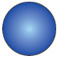	105	0.23	1.2
16	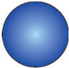	118	0.24	1.0
24	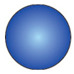	134	0.24	1.0
32	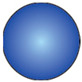	145	0.26	1.2
40	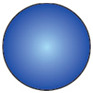	152	0.27	1.1
70	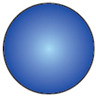	171	0.33	0.8
100	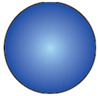	176	0.36	0.7
130	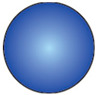	179	0.39	0.8
160	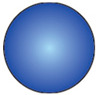	178	0.43	0.9
180	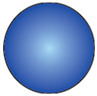	180	0.40	0.8

**Table 4:** SANS results: structural model fit parameters of AY38/G5 prolonged kinetics at *l_c_* = 3.0 (as shown in Figure 5b). The polydispersity of the spherical particles in size is given as PDI. The reduced *χ*^2^ shows the quality of the fit.

Time/min	Shape	Radius/nm	PDI	*χ* ^2^
3	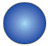	59	0.30	1.3
6	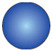	76	0.20	1.4
9	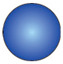	84	0.23	1.3
12	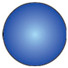	92	0.22	0.9
15	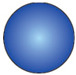	100	0.22	0.9
20	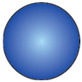	106	0.26	0.9
25	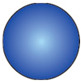	121	0.22	1.1
30	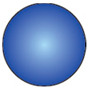	123	0.30	0.8
35	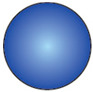	126	0.34	0.9
40	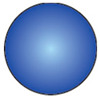	132	0.25	1.0
70	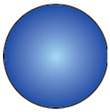	181	0.30	0.9
100	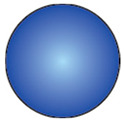	206	0.36	0.7
130	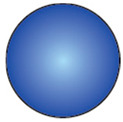	189	0.40	0.8
180	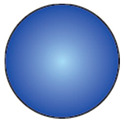	202	0.38	0.9

**Table 5:** Comparison between method A and method B.

Key Features	Pre-Irradiation (Method A)	Post-Irradiation (Method B)
Kinetics	Slow kinetics, ≈3 h	Fast kinetics, <20 min
Irradiation	Pre-irradiated AY38 (*cis* state) before assembly	UV irradiation during self-assembly
Properties	Allows controlled, stepwise formation, ideal for detailed analysis	Captures immediate, rapid transformations

**Table 6:** Summary of the shapes observed, calculated from SLS-SANS data prior to or after the irradiation using pre- and post-irradiation methods for AY38 assemblies with different dendrimer generations at multiple charge ratios. Method A represents multiple steps of the assembly growth until the system stabilizes (t_0_ = 0–30 min, t_m_ = 30–150 min, and t_f_ = > 150 min), whereas method B only shows the start and the end product.

Pre-Irradiation (Method A)					
System ID	*l_c_*	After Irradiation (t_−1_)	t_0_	t_m_	t_f_
AY38/G4	2.0	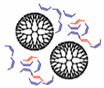	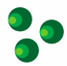	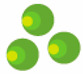	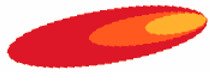
AY38/G5	2.0	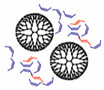	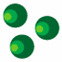	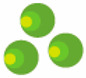	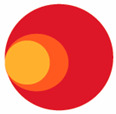
	3.0	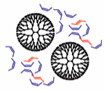	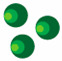	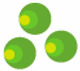	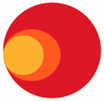
**Post-Irradiation (Method B)**					
**System ID**	***l_c_***	**Before Irradiation**		**After Irradiation**	
AY38/G4	1.5	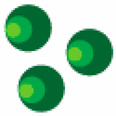		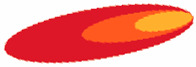	
AY38/G5	1.5	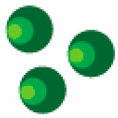		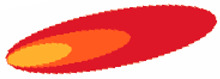	
	2.0	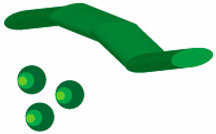		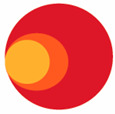	

## Data Availability

The data presented are included in this study. SANS data will be available at https://doi.ill.fr/10.5291/ILL-DATA.9-12-662 (accessed on 30 August 2021), and https://doi.ill.fr/10.5291/ILL-DATA.9-11-2079 (accessed on 28 September 2021).

## Supplementary Materials

The following supporting information can be downloaded at: https://www.mdpi.com/article/10.3390/polym18020190/s1, Figure S1: Comparison of spherical and ellipsoidal model fits to the long-kinetics SANS data of AY38/G4 at *l_c_* = 2.0 for the 30 min acquisition spanning 120–150 min. (**a**) Best fits of the spherical and ellipsoidal form-factor models to the scattering data. (**b**) Corresponding fit residuals; Figure S2: Time evolution of the forward-scattering intensity extrapolated to *q*→0 (*I*_0_) from SANS, used here as a proxy for aggregate mass. The data is shown for AY38/G4 assemblies at *l_c_* = 2.0 and AY38/G5 assemblies at *l_c_* = 2.0 and *l_c_* = 3.0; Figure S3: Direct relation between *I*_0_ and the particle volume V calculated using SANS data for AY38/PAMAM dendrimer assemblies of (**a**) G4, *l_c_* = 2.0, and (**b**) G5, *l_c_* = 2.0 and *l_c_* = 3.0. The dotted lines are to guide the eyes to follow the particle volume increase with *I*_0_; Figure S4: For the AY38/PAMAM dendrimer assemblies, *ζ*-potential values are plotted as a function of particle volume for (a) G4, at *l_c_* = 2.0, (b) G5 at *l_c_* = 2.0, *l_c_* = 3.0 and as a function of particle surface area for (c) G4, at *l_c_* = 2.0, and (d) G5 at *l_c_* = 2.0, *l_c_* = 3.0. The dotted lines are used to guide the eyes to follow the change in *ζ*-potential values when the particle volume and the surface area change. Volumes and areas are computed from SANS best-fit dimensions; Figure S5: Time evolution of the charge (*ζR*) for AY38/G5 assemblies at G5, *l_c_* = 2.0 and *l_c_* =3.0. Size R is taken from SANS fits. The dotted lines are used to guide the eyes to follow the change in charge values when the particle volume changes; Figure S6: Particle volume calculated using fast kinetics SANS results with their fitted data of the AY38/G5 system at *l_c_* = 0.5, 1.5, 2.0, and 3.0; Table S1: SANS Results: Comparison of ellipsoidal and spherical model fit parameters of AY38/G4 long kinetics at *l_c_* = 2.0. The radius of the spherical particles is given as R_min_; in the case of ellipsoidal particles, R_min_ shows two axial radii, and R_maj_ shows the equatorial radius. Polydispersity in size is given as PDI. The reduced *χ*^2^ shows the quality of the fit; Table S2: SANS Results: Structural model fit parameters of AY38/G5 short kinetics at *l_c_* = 3.0. The radius of the cylindrical particles is given as R and the length as L. The polydispersity of the particles is given as PDI. The reduced *χ*^2^ shows the quality of the fit; Table S3: SANS Results: Structural model fit parameters of AY38/G5 short kinetics at *l_c_* = 1.5. The radius of the spherical particles is given as R_min_; in the case of ellipsoidal particles, R_min_ shows two axial radii_,_ and R_maj_ shows the equatorial radius. Polydispersity in size is given as PDI. The reduced *χ*^2^ shows the quality of the fit; Table S4: SANS Results: Structural model fit parameters of AY38/G5 short kinetics at *l_c_* = 2.0. The polydispersity of the spherical particles in size is given as PDI. The reduced *χ*^2^ shows the quality of the fit; Table S5: SANS Results: Structural model fit parameters of AY38/G5 short kinetics at *l_c_* = 3.0. The polydispersity of the spherical particles in size is given as PDI. The reduced *χ*^2^ shows the quality of the fit.
