# Supplementary material for: Light-Induced Structural Evolutions in Electrostatic Nanoassemblies
Source: Polymers (Basel). 2026 Jan 9;18(2):190. doi: 10.3390/polym18020190 (PMC12845469; doi:10.3390/polym18020190)
Supplement: Supplementary file 1 [file polymers-18-00190-s001.zip › polymers-4065518-supplementary.pdf]

Supporting Information

Light-Induced Structural Evolutions in Electrostatic Nanoassemblies

Mohit Agarwal [1,2], Ralf Schweins [2] and Franziska Gröhn [1,*]

[1] Department of Chemistry and Pharmacy, Interdisciplinary Center for Molecular Materials, Friedrich-Alexander Universität Erlangen-Nürnberg, Egerlandstr. 3, D-91058 Erlangen, Germany; mohit.agarwal@fau.de

[2] Institut Laue-Langevin, DS/LSS, 71 Avenue des Martyrs, F-38000 Grenoble, France; schweins@ill.eu

* Correspondence: franziska.groehn@fau.de; Tel.: +49-913-185-20731

Additional Results:

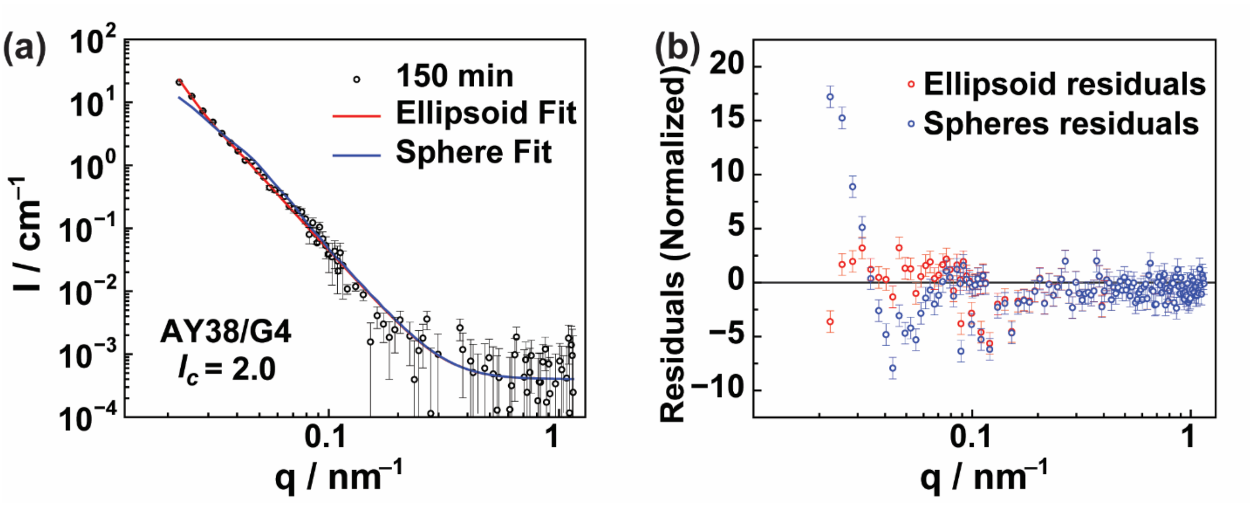

Figure S1. Comparison of spherical and ellipsoidal model fits to the long-kinetics SANS data of AY38/G4 at l_c = 2.0 for the 30 min acquisition spanning 120–150 min. **(a)** Best fits of the spherical and ellipsoidal form-factor models to the scattering data. **(b)** Corresponding fit residuals.

Table S1. SANS Results: Comparison of Ellipsoidal and spherical model fit parameters of AY38/G4 long kinetics at l_c = 2.0. The radius of the spherical particles is given as Rmin; in the case of ellipsoidal particles, Rmin shows two axial radii, and Rmaj shows the equatorial radius. Polydispersity in size is given as PDI. The reduced χ^2 shows the quality of the fit.

Time/min	Shape	R_{min} /nm	PDI (R_{min})	R_{maj} /nm	PDI (R_{maj})	χ^2
150	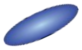	35	0.30	139	0.02	2.0
150	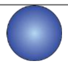	130	0.30			8.2

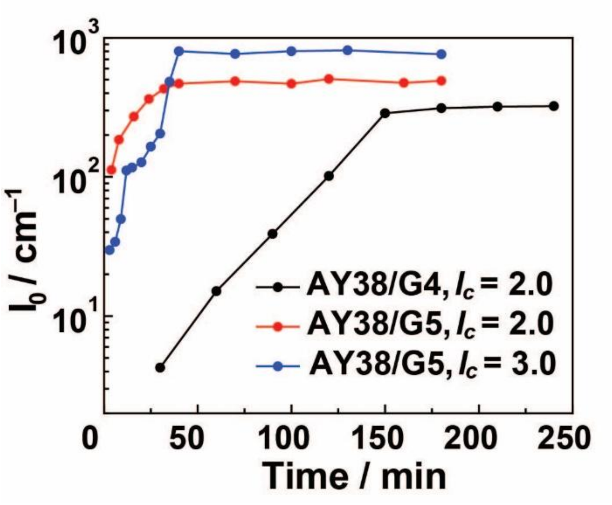

Figure S2. Time evolution of the forward-scattering intensity extrapolated to q→0 (I_0) from SANS, used here as a proxy for aggregate mass. The data is shown for AY38/G4 assemblies at l_c = 2.0, and AY38/G5 assemblies at l_c = 2.0 and l_c = 3.0.

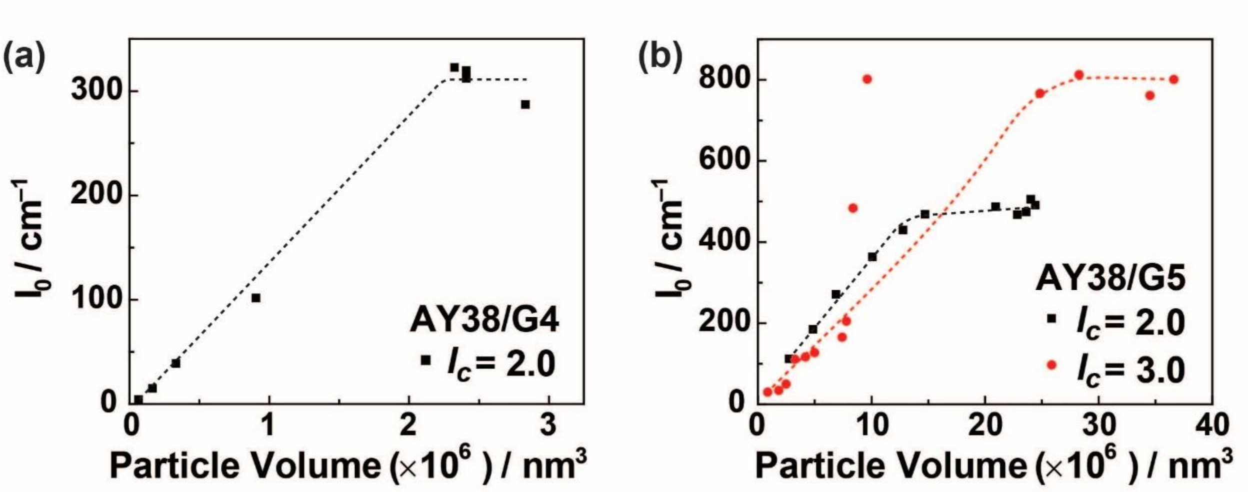

Figure S3. Direct relation between I_0 and the particle volume V calculated using SANS data for AY38/PAMAM dendrimer assemblies of **(a)** G4, l_c = 2.0, and **(b)** G5, l_c = 2.0 and l_c = 3.0. The dotted lines are guide to the eyes to follow the particle volume increase with I_0.

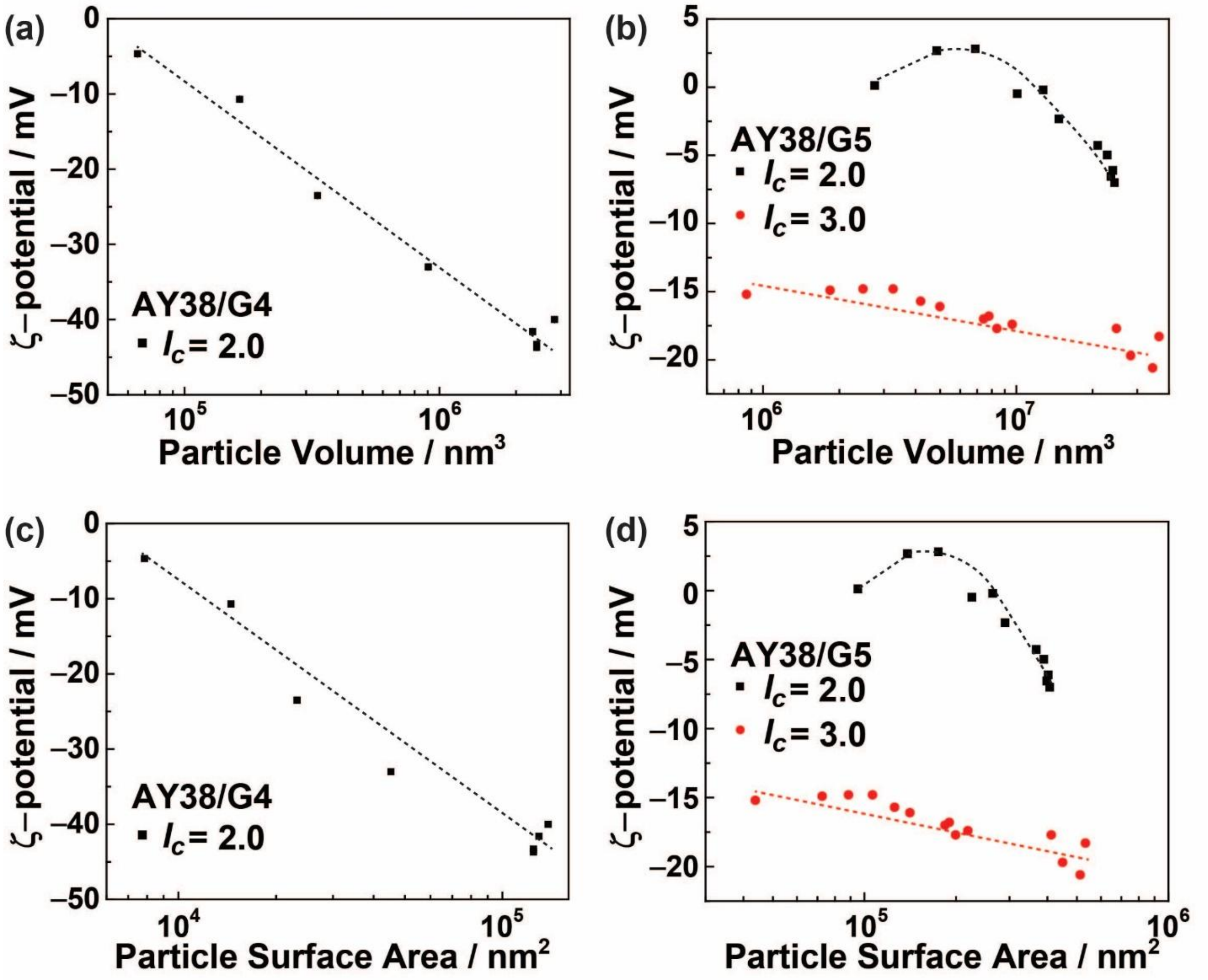

Figure S4. For the AY38/PAMAM dendrimer assemblies, ζ-potential values are plotted as a function of particle volume for (a) G4, at $l_c = 2.0$, (b) G5 at $l_c = 2.0$, $l_c = 3.0$ and as a function of particle surface area for (c) G4, at $l_c = 2.0$, (d) G5 at $l_c = 2.0$, $l_c = 3.0$. The dotted lines are used to guide the eyes to follow the change in ζ-potential values when the particle volume and the surface area change. Volumes and areas are computed from SANS best-fit dimensions.

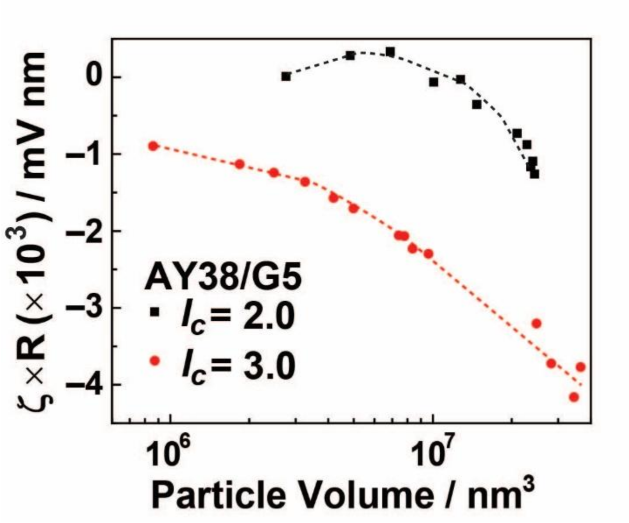

Figure S5. Time evolution of the charge (ζR) for AY38/G5 assemblies at G5, l_c = 2.0 and l_c =3.0. Size R is taken from SANS fits. The dotted lines are used to guide the eyes to follow the change in charge values when the particle volume changes.

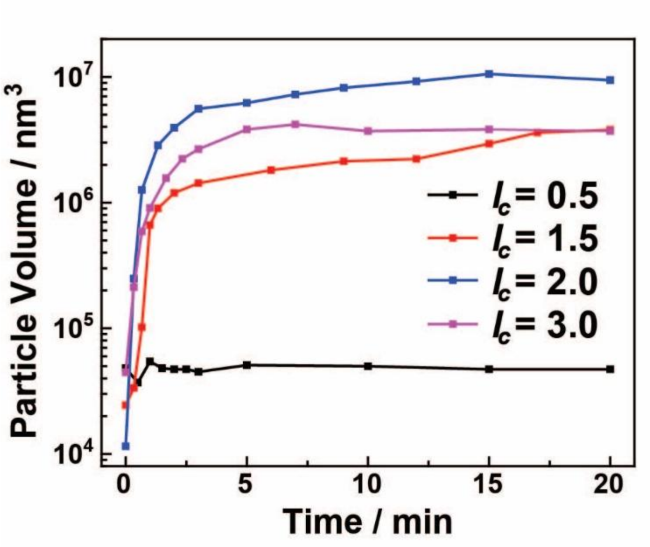

Figure S6. Particle volume calculated using fast kinetics SANS results with their fitted data of AY38/G5 system at l_c = 0.5, 1.5, 2.0, and 3.0.

Table S2. SANS Results: Structural model fit parameters of AY38/G5 short kinetics at $l_c = 3.0$. The radius of the cylindrical particles is given as R and the length as L. The Polydispersity of the particles is given as PDI. The reduced χ^2 shows the quality of the fit.

Time	Shape	R/nm	PDI (R)	L /nm	PDI (L)	χ^2
Before Irradiation	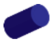	17	0.03	53	0.05	1.6
30 s	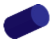	16	0.05	46	0.01	0.9
60 s	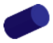	17	0.01	60	0.10	1.3
90 s	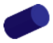	17	0.04	53	0.02	1.1
120 s	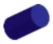	17	0.03	52	0.04	1.1
150 s	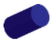	17	0.05	52	0.10	1.3
180 s	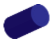	16	0.03	56	0.03	0.9
5 min	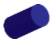	17	0.04	56	0.05	1.5
10 min	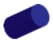	17	0.01	55	0.04	1.6
15 min	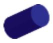	17	0.04	52	0.02	1.9
20 min	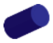	17	0.05	52	0.10	1.9

Table S3. SANS Results: Structural model fit parameters of AY38/G5 short kinetics at $l_c = 1.5$. The radius of the spherical particles is given as R_{min}; in the case of ellipsoidal particles, R_{min} shows two axial radii, and R_{maj} shows the equatorial radius. Polydispersity in size is given as PDI. The reduced χ^2 shows the quality of the fit.

Time	Shape	R_{min}/nm	PDI (R_{min})	R_{maj}/nm	PDI (R_{maj})	χ^2
Before Irradiation	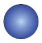	18	0.14			1.5
20 s	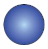	20	0.20			1.3
40 s	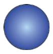	29	0.30			1.3
60 s	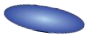	20	0.40	89	0.10	1.2
80 s	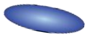	32	0.20	82	0.11	1.2
120 s	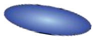	36	0.30	89	0.10	1.4
180 s	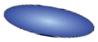	44	0.20	88	0.11	1.9
6 min	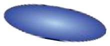	50	0.20	93	0.02	1.6
9 min	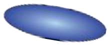	47	0.11	104	0.01	1.1
12 min	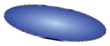	51	0.20	102	0.02	0.8
15 min	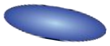	54	0.33	114	0.10	0.9
17 min	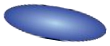	55	0.30	125	0.10	1.3
20 min	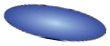	57	0.20	126	0.02	1.0

Table S4. SANS Results: Structural model fit parameters of AY38/G5 short kinetics at l_c = 2.0. The polydispersity of the spherical particles in size is given as PDI. The reduced χ^2 shows the quality of the fit.

Time	Shape	Radius/nm	PDI	χ^2
Before Irradiation	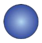	14	0.23	1.9
20 s	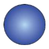	39	0.03	1.3
40 s	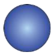	67	0.05	1.6
80 s	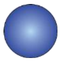	88	0.20	1.6
120 s	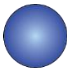	98	0.20	1.5
180 s	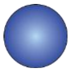	110	0.11	1.6
5 min	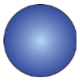	114	0.22	2.1
7 min	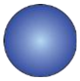	120	0.18	2.2
9 min	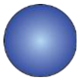	125	0.20	2.3
12 min	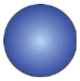	130	0.03	2.5
15 min	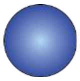	136	0.04	1.7
20 min	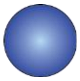	131	0.06	1.8

Table S5. SANS Results: Structural model fit parameters of AY38/G5 short kinetics at l_c = 3.0. The polydispersity of the spherical particles in size is given as PDI. The reduced χ^2 shows the quality of the fit.

Time	Shape	Radius/nm	PDI	χ^2
Before Irradiation	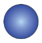	22	0.03	0.9
20 s	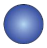	37	0.03	1.3
40 s	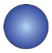	52	0.05	1.3
60 s	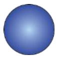	60	0.10	1.2
100 s	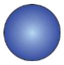	72	0.28	1.6
140 s	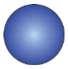	81	0.20	1.1
180 s	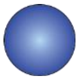	86	0.22	2.1
5 min	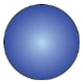	97	0.20	1.0
7 min	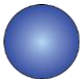	100	0.11	1.0
10 min	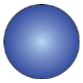	96	0.26	0.9
15 min	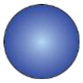	97	0.16	1.5
20 min	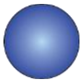	96	0.21	0.9
